# Uncovering the Molecular Mechanism of Actions between Pharmaceuticals and Proteins on the AD Network

**DOI:** 10.1371/journal.pone.0144387

**Published:** 2015-12-09

**Authors:** Shujuan Cao, Liang Yu, Jingyuan Mao, Quan Wang, Jishou Ruan

**Affiliations:** 1 College of Mathematical Sciences and LPMC, Nankai University, Tianjin, PRC; 2 Internal Medicine for the Heart, Tianjin University of Traditional Chinese Medicine, Tianjin, PRC; 3 National Laboratory of Macromolecules, Institute of Biophysics, Chinese Academy of Science, Beijing, PRC; 4 High Throughput Molecular Drug Discovery Center, Tianjin International Joint Academy of Biomedicine, TEDA, Tianjin, PRC; 5 State Key Laboratory for Medical Chemical and Biology, Nankai University, Tianjin, PRC; Institute of Health Science, CHINA

## Abstract

This study begins with constructing the mini metabolic networks (MMNs) of beta amyloid (Aβ) and acetylcholine (ACh) which stimulate the Alzheimer’s Disease (AD). Then we generate the AD network by incorporating MMNs of Aβ and ACh, and other MMNs of stimuli of AD. The panel of proteins contains 49 enzymes/receptors on the AD network which have the 3D-structure in PDB. The panel of drugs is formed by 5 AD drugs and 5 AD nutraceutical drugs, and 20 non-AD drugs. All of these complexes formed by these 30 drugs and 49 proteins are transformed into dyadic arrays. Utilizing the prior knowledge learned from the drug panel, we propose a statistical classification (dry-lab). According to the wet-lab for the complex of amiloride and insulin degrading enzyme, and the complex of amiloride and neutral endopeptidase, we are confident that this dry-lab is reliable. As the consequences of the dry-lab, we discover many interesting implications. Especially, we show that possible causes of Tacrine, donepezil, galantamine and huperzine A cannot improve the level of ACh which is against to their original design purpose but they still prevent AD to be worse as Aβ deposition appeared. On the other hand, we recommend Miglitol and Atenolol as the safe and potent drugs to improve the level of ACh before Aβ deposition appearing. Moreover, some nutrients such as NADH and Vitamin E should be controlled because they may harm health if being used in wrong way and wrong time. Anyway, the insights shown in this study are valuable to be developed further.

## Introduction

Currently, the causes of Alzheimer’s Disease (AD) remain quite unclear and no safe and effective drug may stop or reverse the progression of AD although many hypotheses have been proposed including cholinergic-, tau protein-, amyloid- hypotheses [1–3] and many risk factors have been identified including apolipoprotein E (APOE) [4–5], mutations of triggering receptor expressed on myeloid cells 2 (TREM2) [6–7], susceptibility loci [8], viral [9], age-related myelin breakdown [10], dys-homeostasis [11], diabetes and cardiovascular risk factors [12], *etc*. Moreover, the epidemiological studies during 2005–2007 have showed that the prevalence of AD is increasing [13] while pharmaceuticals emerged as a new risk factor [14]. Therefore, it is appealing to discover safe and effective pharmaceutical treatments and to develop non-invasive imaging agents to detect AD. In addition, it is also important to understand the mechanism of pharmaceutical products and their effects on the prevalence of AD. This requires new insights into molecular mechanism of action between the available pharmaceuticals and the proteins on AD network. For simplicity, in the rest of this paper we only use the word “drug” which could means ligand, compound, or pharmaceutical.

There are three typical strategies to design drugs: (1) Choosing converting enzymes of the stimulus as the target in order to down-regulate the level of the stimulus; (2) Choosing cleaning enzymes as the target in order to up-regulate the level of the stimulus; and (3) Choosing the receptor as the target in order to prevent the specific function induced by the given stimulus from docking on a receptor. Some drugs may dramatically alter the level of the specific stimulus, and therefore they are welcomed by both doctors and patients. Nevertheless, clinical evidences show that almost all metabolic diseases cannot be completely cured with drugs, although the levels of the markers can be regulated to the expected level by these drugs. How to solve this puzzle is the main goal of drug design. An insight has newly emerged from industry which requires that multiple mechanisms of a disease should be covered when designing a particular drug (see A BioMAP. Drug Discovery Case Study). However, how to cover many mechanisms simultaneously has not yet been elucidated.

Since the molecular mechanism for conventional drug designing is increase or decrease the level of a stimulus. In practice, a disease may have more stimuli at the same time. For example, brain natriuretic peptide (BNP), angiotensin II (Ang-II or AII for short), and aldosterone are markers related to heart failure (HF) and hypertension (HT). Historically, while one drug is used to decrease the level of Ang-II, it can increase the level of BNP at the same time. Therefore a double inhibitor of NEP and ACE was proposed and Omapatrilat, Alatrioprilat, Sampatrilat and Gemopatrilat were thought to be the most potent double inhibitors [15]. After substantial investigating, they were found to have failed the phase-III trials because the side-effects of these double inhibitors are stronger than either the NEP inhibitor/ACE inhibitor individually or a combination of them [16]. This reveals that artificially designed multiple inhibitors are both difficult and highly risky [17]. In this study, we will show that our computational results suggest multiple inhibitors exist if we discover them in accordance with their natural tendency rather than through our own design.

We organize the rest of this paper as follows. Firstly, we show how to construct the AD network based on basic hypotheses and risk factors, how to form the panel of proteins and the panel of drugs, and then transform all complexes formed by the drugs and proteins in the panels into dyadic arrays by using ILbind [18] and Vina, the newest version of Autodock [19]. Secondly, we model the statistical classification by learning from 30 complexes formed by the given drugs and their targets. We then divide all 1,470 complexes into 7 groups of H_0_-H_6_ according to their dyadic arrays, where the complexes in H_0_ are highly recommended while those in H_6_ are highly rejected. Obviously, the complexes Amiloride-Insulin degradation enzyme (AMR-IDE) and Amiloride- neutral endopeptidase (AMR-NEP) are located at the top corner of H_1_ and therefore their positions are the worst cases relative to most of complexes in H_0_ or H_1_. The IC_50_ results show that AMR strongly inhibits IDE and weakly inhibits NEP, which suggests that drug binds the protein is a high probability event for most complexes in H_0_ or H_1_. Thirdly, we discuss on filtering some false positive complexes according to the function of the binding sites and binding position [20–21]. Finally, we draw some potential conclusions.

## Materials and Methods

### Constructing the AD network

Although this study focuses on AD, our proposed framework is applicable to all metabolic diseases. We begin with drug designing for metabolic diseases. Conventionally, investigating a mechanism of a metabolic disease constitutes three main steps: (1) picking stimuli that are strongly related to the given disease, based on clinical cases; (2) among all strongly related stimuli, selecting a measureable and stable stimulus, which has both high sensitivity and specificity for diagnosing and managing the disease, as a biomarker; and (3) for each selected stimulus, finding its precursor, the enzymes that convert the precursor to the stimulus, the receptors of the stimulus and their associated functions, or the enzymes for cleaning the stimulus.

**Fig 1:**
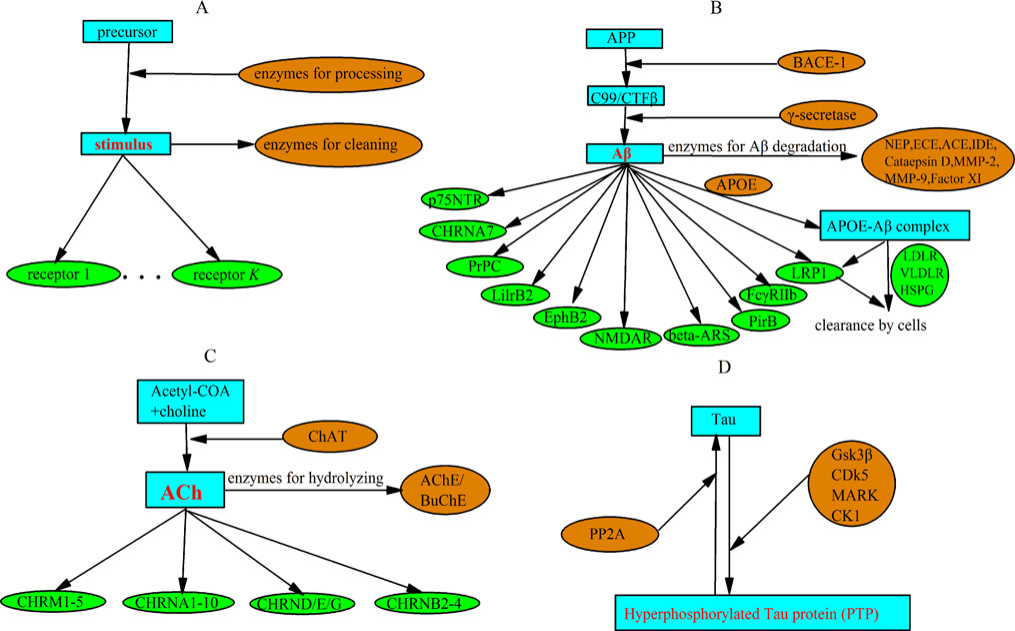
(A) The general form of MMN of a stimulus. (B) The MMN of Aβ. (C) The MMN of ACh. (D) The MMN of tau protein. The stimuli (middle products), enzymes, and receptors are highlighted with red, orange, and green colors, respectively.

In order to explore ways of integrating the related mechanisms, we construct the mini metabolic network (MMN) of a given stimulus. For a given stimulus, the center of an MMN should consists of the precursors, enzymes for converting the stimulus from the precursor, receptors for accepting the stimulus, and enzymes for cleaning the stimulus. Its standard form is shown in **Fig 1A**. Of course, in practice we can obtain many non-standard variants of an MMN because the precursors, the converting enzymes, and the cleaning enzymes can be regulated by other enzymes, and the receptors may be affected by the endogenic antagonists. Especially, many receptors are GPCR proteins, and therefore have tails composed of G-protein and enzymes. Beta-amyloid (Aβ), acetylcholine (ACh) and phosphorylated tau protein (PTP) are three markers related to AD. We begin with constructing the MMNs of Aβ, Ach, and PTP, as show in **Fig 1B, 1C and 1D**. For detail, see S1 File.

**Fig 2:**
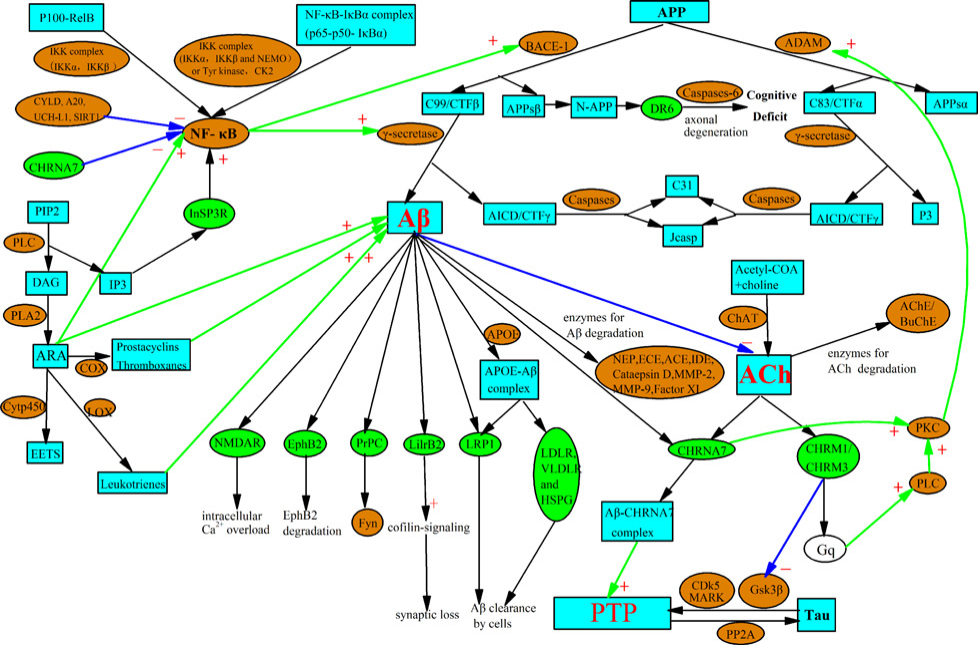
The AD network generated based on three mini metabolic networks. For clearly understanding this graph, we state the nodes and edges as follows: enzymes, stimuli, precursors and receptors are the nodes. In which, enzymes are shown by orange ovals, stimuli and their precursors are shown by cyan boxes, and receptors are shown by green ovals. The edges are consisted of three kinds of arrows. In which, green arrows with “+” indicates the up-regulating or activating relationship, while blue arrows with “–” means the down-regulating or inhibiting relationship. The black arrows are the normal up- and down- stream relationship.

In order to generate the AD network based on the three MMNs, we require their cross-talk information and additional auxiliary information mined from the literature, some other stimuli which do not appear in the three MMNs but activate or inactivate the enzymes of MMNs. For example, NF-κB activates BACE-1 [22–23], and PKC activates ADAM [24]. The interaction between arachidonic acid (AA) and Aβ follows previous work [25], as does the interaction between protein tau and Aβ [26–27], the interaction between acetylcholine and Aβ [28–32], *etc*. For details, see Text A in S1 File of this paper. We construct an AD network which is much larger than the union of three MMNs, as shown in **Fig 2**.

### Panel of proteins and panel of drugs

We sort out these enzymes and receptors on the AD network to form the panel of proteins if they have a 3D-structure (partial or entire) in PDB. Consequently, 49 enzymes/receptors with partially or entirely known structure in PDB are selected into the panel from the AD network, and are listed in **Table A in** S1 **File**. Each enzyme/receptor in the panel has more than one versions of 3D-structure in PDB. Especially, many enzymes/receptors have a large amount of versions in PDB, for example, the number of the versions of BACE-1 in PDB is more than 200. For these cases, we will use the alignment algorithm to select a few versions which are the best representatives of the 3D-structure of an enzyme or a receptor.

In order to show the effects of the AD drugs through using computer-aided approaches, we also need to construct a panel of drugs composed of AD drugs and non-AD drugs. We select 5 AD drugs and 5 nutraceutical drugs, including (1) Donepezil (E20), Galantamine (GNT), Tacrine (THA), and Huperzine A (HUP), which originally are AChE inhibitors [33–34]; (2) Memantine (377) is the unique antagonist of NMDAR [34]; and (3) the other 5 nutraceutical drugs are randomly selected from Drugbank. For example, NADH (NAI) is the nutraceutical [35], which is used to improve the function of the brain and central nervous system. For the non-AD drugs, we select 9 diabetes drugs and 11 drugs used for heart failure. Additionally, we add amiloride (AMR) as the referential drug, and it is not included in the panel. All 31 drugs are listed in **Table B in** S1 **File**.

### The data transformed from all complexes formed by drug and protein in the panels

Because the total number of possible drug-protein complexes formed by drugs and proteins in panels is 1470 (= 30*49), it is a time-consuming and money-consuming work if we check all complexes with wet-lab. Therefore, we employ dry-lab to elucidate the potential relationships. In this subsection, we will use two tools, ILbind [18] and Vina, the newest version of AutoDock [19], to perform the task. Despite that ILbind is a potent tool to provide plentiful messages, it just fits to process single-chain of a protein. If we use ILbind individually, some actual binding relationships between drugs and proteins will be missed. This is because some medical pockets are the clefts formed by two or more chains and then ILbind cannot find it but Vina may correctly find it.

We transform all 1,470 complexes to the dyadic array as the form (*x*_1_, *x*_2_), in which *x*_1_ is the value of the similarity output from ILbind, and *x*_2_ is the minimal free energy output from VINA for each complex formed by a drug and a protein in the panels. To fit in a single page, we present the data of 10 AD drugs and the 49 proteins in **Table C in** S1 **File**, the data of 9 diabetes drugs and 49 proteins in **Table D in** S1 **File**, and the data of 12 HF drugs (include AMR as the referential drug) and 49 proteins in **Table E in** S1 **File**, respectively.

### Prior knowledge about the values of similarity and free energy

In order to understand the meanings shown in Tables C, D and E in S1 File, we begin with learning prior knowledge from the values of similarity and free energy based on the panel of drugs. For each drug, we search for the protein which is the original drug target, called its “target”. Each complex of drug and its target can then be converted to a dyadic array (*x*_1_, *x*_2_). We show the data in **Table** 1.

**Table 1:** The values of similarity and free energy based on 30 drugs on their targets. In which *x*_1_ is the value of similarity output from ILbind, and *x*_2_ is the minimal free energy output from VINA for each complex formed by a drug and a protein ranging to the panels

*No*.	*Drug*	*Target*	*PDB-id*	*x*_1_	*x*_2_	*No*.	*Drug*	*Target*	*PDB-id*	*x*_1_	*x*_2_
1	E20	AChE	4bdt	0.9	-7	16	LF7	DPP-4	*2qtb*	0.91	-7.2
2	GNT	AChE	4bdt	0.9	-7.5	17	ACR	MGAM	3ctt	0.92	-8
3	THA	AChE	4bdt	0.91	-6.5	18	MIG	MGAM	3ctt	0.92	-5.7
4	HUP	AChE	4bdt	0.91	-9.6	19	X8Z	ACE	1o8a	0.88	-5.8
5	CHT	AChE	4bdt	0.71	-3.6	20	X93	ACE	1o8a	0.91	-8.7
6	LPA	LIPT1	3a7u	0.86	-4.5	21	LPR	ACE	1o8a	0.91	-7.7
7	PSF	PRKCA	4dnl	0.8	-5.2	22	06X	CA1	1azm	0.55	-6.2
8	NAI	UDPGDH	3prj	0.88	-8.7	23	FUN	CA2	1fsn	0.88	-7.5
9	VIV	PRKCA	3iw4	0.71	-7.8	24	TLS	AT1	3vn2	0.75	-8.4
10	BRL	PPARγ	2xyw	0.88	-8.5	25	TIM	ADRB1	2y00	0.88	-6.2
11	P1B	PPARγ	2xyw	0.88	-9.2	26	SNP	ADRB1	2y00	0.51	-6.3
12	T22	DPP-4	2qtb	0.91	-7.4	27	CVD	ADRB1	2y00	0.88	-7.5
13	356	DPP-4	2qtb	0.91	-8.2	28	2TN	ADRB1	2y00	0.53	-7.2
14	715	DPP-4	2qtb	0.91	-8.3	29	CLU	CYP2D6	3qm4	0.64	-6.9
15	BJM	DPP-4	2qtb	0.91	-7.7	30	377	NMDAR	3jpw	0.49	-5.9

Based on these 30 dyadic arrays, we have the mean *m* and standard deviation σ. The derived data regarding similarity and free energy are listed as show in Table 2.

**Table 2:** The derived data regarding similarity and free energy based on mean *m* and standard deviation *σ*.

	σ	*m*-3σ	*m*-2σ	*m*-σ	*m*	*m*+σ	*m*+2σ	*m*+3σ
similarity	0.14	0.40	0.54	0.68	0.82	0.96	-	-
free energy	1.4	-11.4	-10.0	-8.6	-7.2	-5.8	-4.4	-3.0

From the dyadic arrays of 30 complexes, we may find that the distribution of values of similarity is not normal. Typically, the values of similarity are distributed in [0.4, 0.96]. Specifically, 3 samples are located in [0.4, 0.54], 2 samples in [0.54, 0.68], and 25 samples in [0.68, 0.96]. Regarding the benchmark set, the sensitivity of similarity is 0.833 if the low-bound is 0.68, or 0.9 if the low-bound id 0.54. Similarly, the values of free energy are not normally distributed either. Typically, 1 sample belongs to interval [-4.4, -3.0], 4 samples belong to [-5.8, -4.4], and the rest 25 samples belong to [-10, -5.8]. In other words, the sensitivity of free energy is 0.833 if the up-bound is -5.8, or 0.967 if the up-bound is -4.4. This indicates that the mean and the standard deviation of similarity or the free energy will be slightly changed as the size of samples increases or the outlier samples are discarded. In the rest part, we choose 0.68 as the threshold of similarity and -5.8 as the threshold of free energy.

**Fig 3:**
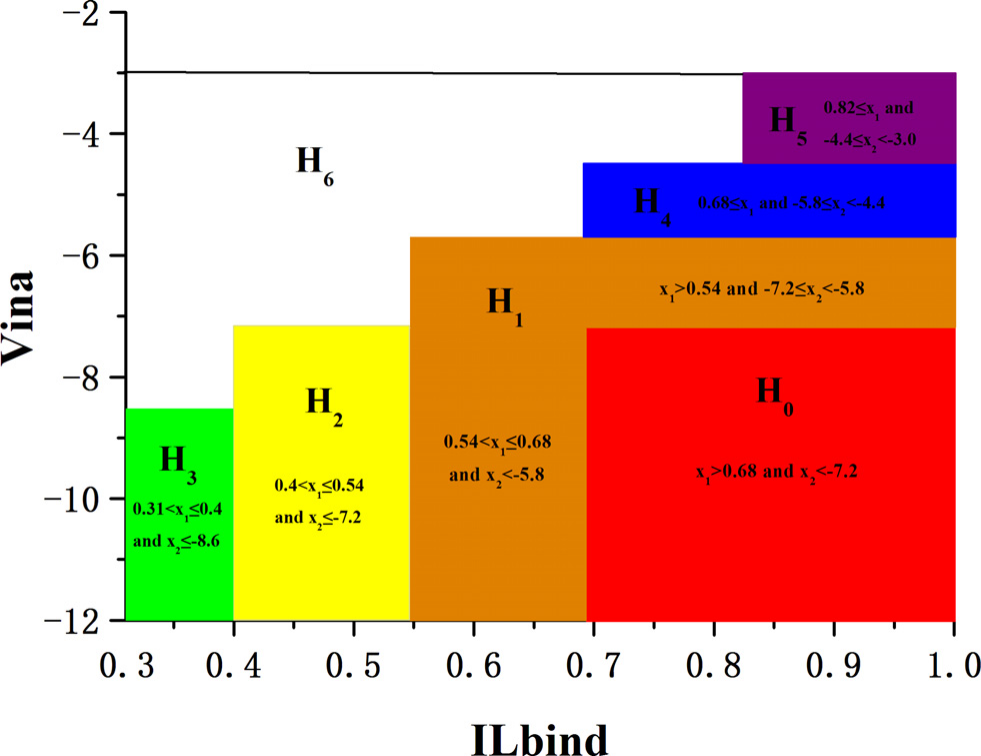
The seven areas (H_0_- H_6_) of complexes to be recommended or rejected. H_0_ is colored with red, H_1_ is colored with orange, H_2_ is colored with yellow, H_3_ is colored with green. H_4_ is colored with blue, H_5_ is colored with purple, and H_6_ is blank.

In practice, the values of free energy are quite scattered. The value of free energy in [-10, -7.2] implies that the affinity of the ligand binding to the target cannot be ignored. Moreover, if the value of free energy is in [-11.4, -10.0], or less than -11.4, we should consider the value of similarity when evaluating whether or not the ligand may bind to the target, even though the value may be quite small. These cases are due to the fact that drugs do not bind to one domain, but rather to the clefts formed by two or more domains, which constitutes the most important drug targets for discovering drugs. Furthermore, for a small ligand which is more smaller relative to the pocket or a part of a drug binding to the pocket, its similarity is greater than 0.68, but its free energy is greater than -5.8. Based on the mean, standard deviation, and the derived data about similarity and free energy, we classify the (*x*_1_, *x*_2_)-plan into seven areas as shown in **Fig 3**. Typically, area H_0_: *x*_1_ > 0.68 and *x*_2_ < -7.2, is colored with red. Area H_1_: 0.54 < *x*_1_ ≤ 0.68 and *x*_2_ < -5.8, or 0.54 < *x*_1_ and -7.2 ≤ *x*_2_ ≤ -5.8, it is the boundary zone of H_0_ and colored with orange. Area H_2_: 0.44 < *x*_1_ ≤ 0.54 and *x*_2_ ≤ -7.2, is colored with yellow. Area H_3_: 0.31 < *x*_1_ ≤ 0.4 and *x*_2_ ≤ -8.6, is colored with green. Area H_4_: 0.68 ≤ *x*_1_ and –5.8 < *x*_2_ < -4.4, is colored with blue. Area H_5_: 0.82 ≤ *x*_1_ and –4.4 ≤ *x*_2_ < -3.0 is colored with cyan, and the blank area is named H_6_.

According to the dyadic arrays of the 30 complexes shown in **Table** 1 and the divisions in **Fig 3**, we find 17 samples in H_0_, 4 samples in H_1_, 1 sample in H_2_, 0 sample in H_3_, 4 samples in H_4_, and 0 sample in H_5_. In addition, 4 complexes which fall in H_6_ should be rejected, this means the correct rate of this decision is 0.867 on this benchmark set. However, this benchmark set is not adequate because it has no negative samples. In general, a queried set of complexes often has no prior knowledge. We wish to construct a benchmark set which contains enough positive and negative samples so that we could estimate the rate of false positive and the rate of false negative.

Applying the same rules of classification shown in **Fig 3**, we classify these 1,470 complexes based on the dyadic arrays shown in Tables C, D and E in S1 File into 7 classes: H_0_, H_1_, H_2_, H_3_, H_4_, H_5_ and H_6_, and the distribution of complexes in different areas are listed in **Table** 3.

**Table 3:** Distribution of complexes falling into H_0_-H_6_ in Tables C, D and E in S1 File.

NO. of set	H_0_	H_1_	H_2_	H_3_	H_4_	H_5_	H_6_
Table C	67	123	26	1	0	0	273
Table D	46	106	55	13	1	0	220
Table E	26	182	52	1	3	0	275
Total	139	411	133	15	4	0	768

More than half of all complexes (768 of 1,470) can be rejected directly as they fall in H_6_. The sum of the number of H_0_ and H_1_ is 550, which is still too many to be tested one by one with a wet-lab by our group alone. Since complex AMR-IDE with dyadic array (*x*_1_, *x*_2_) = (0.55, -7.0) and complex AMR-NEP with dyadic array (*x*_1_, *x*_2_) = (0.6, -6.9) are located in the top corner of H_1_, we can get more confidence to show that the drugs bind to the corresponding proteins with high probability for these 550 complexes through testing that AMR binds to both IDE and NEP with wet-lab.

### Test of AMR-IDE and AMR-NEP with wet-lab

The amiloride has two analogs including 5-(N, N-Dimethyl) amiloride hydrochloride and amiloride HCl dehydrate. In order to avoid the unfair output resulted by selection, two analogs are used in the test. In other words, we confirm that amiloride binds to IDE, and amiloride binds to NEP if both 5-(N, N-Dimethyl) amiloride hydrochloride and amiloride HCl dehydrate may bind to IDE and NEP, respectively.

In order to ensure the correctness of the operations of the wet-lab for showing amiloride binding to IDE, we choose bacitracin A as the referential drug since it was reported as a good IDE inhibitor, and its value of IC_50_ is 100μM. With the same reason, we choose DL-Thiophan as the referential drug for showing amiloride binding to NEP. It was reported that DL-Thiophan is a good NEP inhibitor, and its value of IC_50_ is 0.189 μM. We show the data of these two experiments below, while the simulations for the six cases are depicted in the **Figs A-F in** S1 **File**.

From Table 4, we first confirm that our experimental operations are reliable through comparing the difference between the computed value IC_50_ = 79.25μM and the reported value IC_50_ = 100μM for Bacitracin A binding to IDE, and the difference between the computed value IC_50_ = 0.189μM and the reported value of IC_50_ = 0.0047μM for DL-Thiophan binding to NEP. Then we may conclude that amiloride strongly inhibits IDE because the inhibition rate, the quenching efficiency and value of IC50 for 5-(N, N-Dimethyl) amiloride hydrochloride and amiloride HCl dehydrate binding to IDE are better than those of Bacitracin A. Meanwhile we may also conclude that amiloride weakly inhibits NEP because the corresponding data of 5-(N, N-Dimethyl) amiloride hydrochloride and amiloride HCl dehydrate meet the threshold, while these data are not better than that of DL-Thiophan. As a consequent, AMR is not only the diuretic, but also a double inhibitor of NEP and IDE. If this unexpected conclusion is utilized for hypertension associated diabetes II, the value is a million times more than the initial cost (60,000 RMB). Moreover, the wet-lab tests may enable us to confidently conclude that the drug binds to the protein for each complex in H_0_ or H_1_.

**Table 4:** The inhibition rate and quenching efficiency and value of IC50 for 5-(N, N-Dimethyl) amiloride hydrochloride and amiloride HCl dehydrate binding to IDE and NEP compared with its controlling drug Bacitracin A and DL-Thiophan, respectively.

*No*.	*Compound*	*inhibition rate*	*quenching efficiency*	*IC50/μM*	*Reported value*
1	Bacitracin A	77.08%	-7.05%	79.25	100μM
2	5-(N,N-Dimethyl) amiloride hydrochloride	93.57%	54.77%	178.5	none
3	Amiloride HCl dihydrate	95.02%	-6.64%	214	none
1	DL-Thiophan	102.01%	-4.56%	0.189	4.7nM
2	5-(N,N-Dimethyl) amiloride hydrochloride	80.96%	54.77%	829	none
3	Amiloride HCl dihydrate	65.21%	-6.64%	214	none

### Statistical decision to recommend the complex to be validated with wet-lab

In order to definitively confirm whether or not the drug binds to a protein, the wet-lab test is necessary. However, we should assign the limited resources to test the most possible complexes. Therefore, a good recommendation based on the statistical decision is needed.

#### Decision

We strongly recommend the complexes in H_1_ or H_0_ for the wet-lab test. For complexes in H_2_-H_5_, we moderately recommend the wet-lab test. For complexes in H_6_, we do not recommend the wet-lab test. Even though the complexes in H_1_ or H_0_ have a higher probability which means that the drug could bind to the corresponding proteins, we still need to filter the false positive complexes from H_1_ or H_0_. Therefore, we should consider the function of the binding site, the position of the drug binding to the protein, *etc*. [20–21]. We propose a procedure for filtering false positive complexes as follows:

**Step 1**. For a candidate complex determined by statistical decision, the protein (enzyme/receptor) in the complex may have more than one intrinsic ligand, and their binding sites on this protein can be recovered according to the data shown in PDB. If the binding sites of the drug recommended by Vina do not coincide with (nor are near to) one of these positions of the intrinsic ligands, then we do not recommend the wet-lab test for the complex. Otherwise, we continue on to step 2.
**Step 2**. If one of the binding sites of the drug is the same as the pocket of an intrinsic ligand, we also need to retrieve the pocket containing the intrinsic ligand. If the pocket has no function, then we do not recommend the wet-lab test. Otherwise, we go to step 3.
**Step 3**. If the drug binds to a functional pocket that we desire, then we still need to determine whether or not the binding pose is acceptable by checking the four computable features [21]. If acceptable, we then go to step 4. Otherwise, we do not recommend the wet-lab.
**Step 4**. Even if the drug in a complex has passed steps 1–3 in a fixed binding position, we further need to check whether the small-interference of the given position is still adequate. If many positions within the neighborhood of the given position [20] may also pass step 3, then we recommend the wet-lab for the complex. Otherwise, we do not recommend.

**Fig 4:**
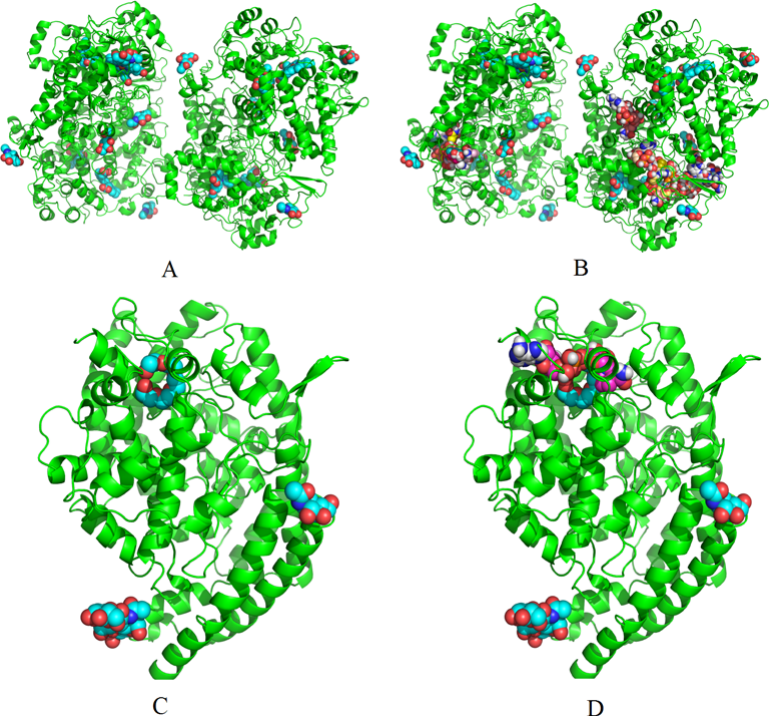
(A) COX-2 complexes in the NSAIDs only. (B) COX-2 complexes in the NSAIDs and NAI. (C) ACE in complex with the intrinsic ligands only. (D) ACE in complex with the intrinsic ligands and NAI.

Each step of the above procedure for filtering false positive complexes may have the chance to filter some false candidates. For example, the crystal structure of complexes formed by SC-558, flurbiprofen and indomethacin binding to COX-2 were obtained in PBD, respectively. Loading these three intrinsic ligands on the same crystal structure (4COX in PDB), we obtain **Fig 4A**. Loading the NAI molecules onto all of the binding sites recommended by Vina, we obtain **Fig 4B**. Comparing Fig 4A and 4B, we may find that binding sites recommended by Vina do not come near the pockets of the three intrinsic ligands. Therefore, the NAI-COX2 complex will be rejected for further checking via the wet-lab at the first step, although the dyadic array of NAI-COX2, (*x*_1_, *x*_2_) = (0.83, -11.6) is quite attractive. ACE possesses three intrinsic pockets to receive the ligand CAPTOPRIL (**Fig 4C**), and the pocket that may receive both CAPTOPRIL and NAI does not receive peptide AII (**Fig 4D**); therefore, the NAI-ACE complex will be rejected for testing via the wet-lab at the second step. The choline is a component of acetylcholine and cannot bind AChE and BHCE as fully as acetylcholine. As a consequence it will be rejected at the third step. HEM is a ligand that binds with HIV-1 protease at just one position. Any small interference will make it unable to enter, and thus it will be rejected at the forth step.

Although the above procedure for filtering false positive complexes has lower cost than the wet-lab test, it still will take too much human labor if we want to falter all false positive complexes from these 550 complexes. Next, we only filter a subset of complexes we have to involve, and we assume that drug may bind to the proteins for each of these complexes which had passed the filtering program.

## Results

### Implications from the data in Table C in S1 File

Checking Table C in S1 File, we may find that enzymes AChE and BCHE are inhibited by E20, GNT, THA and HUP because the dyadic arrays are (0.9, -8.2), (0.9, -8.8), (0.9, -8.0), (0.9, -9.5); (0.9, -8.4), (0.9, -8.4), (0.9,-7.7) and (0.9, -8.1), respectively. From this angle, we agree that E20, GNT, THA and HUP may improve the level of ACh. However, since these 4 drugs cross the blood-brain barrier (BBB) [36], we conclude they are useless to improve the level of ACh. It is against the original design purpose, but they still benefit to AD. The reasons are stated as below:

On one hand, each of E20, GNT, THA and HUP may paralyze the entire MMN of ACh. In fact, E20-ChAT in H_0_, E20-CHRM2, E20-CHRNA4, E20-CHRNB2 in H_1_, and they cannot be filtered by filtering program, therefore we may conclude that E20 inhibits the enzyme ChAT, and blocks most receptors of ACh. In the same way, we may show that GNT, THA and HUP also paralyze the entire MMN of ACh. This implies that E20, GNT, THA and HUP are not only useless to improve the level of ACh, but also do not response the exogenous infusion of ACh.

On the other hand, E20, GNT, THA, and HUP may paralyze the MMN of Aβ. We only use E20 as an example for illustration. In fact, (1) E20-BACE-1 is in H_0_ and (2) E20-NEP, E20-ECE, E20-ACE, E20-IDE are in H_0_; E20-MMP-2 is in H_1_, E20-Cataepsin D, E20-MMP-9, and E20-Factor XI are in H_2_. This result implies that the level of Aβ is kept in initial level without any changing if one of E20, GNT, THA and HUP is used and therefore do not change the state of Aβ deposition.

377 may not block NMDAR because the dyadic array of 377-NMDAR falls into H_6_. It suggests that 377 may not be the selective blocker of NMDAR. Therefore, the role of 377 is not same as the original role shown in instructions. Moreover, since 377-ChAT is in H_1_, we may believe that 377 more possible is the inhibitor of ChAT. It suggests that 377 may not block NMDAR but weaken the activity of the producer ChAT.

Phosphatidylserine (PSF), choline (CHT), Lipoic Acid (LPA), Vitamin E (VIV), and NAI are approved nutraceuticals, rather than drugs. From Table C in S1 File, we see that PSF, CHT, and LPA are not likely to inhibit any of the 49 proteins. However, the data of VIV and NAI are of interest. For the 49 complexes formed by NAI and 49 proteins, 30 complexes in H_0_, 12 complexes in H_1_, and 7 complexes in the H_6_. Filtering these 42 complexes using filtering program, we can conclude that NAI can paralyze both MMNs of ACh and Aβ. Similarly, we get the same results for VIV. Moreover, both NAI and VIV cross the BBB [37–38], it means roles of NAI and VIV similar to the role of E20, GNT, THA, and HUP. Hopefully, NAI is a natural multiple inhibitors for multiple enzymes among A20, ACE, AChE, BACE1, BCHE, ChAT, CYLD, CYP1A1, GSK3β, IDE, MARK, MM-2, NEP, and PP2A. Overall, the role of NAI should be studied further. It shows an example to find the double or multiple inhibitors in natural way.

### Implications of the data in Table D in S1 File

From Table D in S1 File, we may find that Acarbose (ACR), Pioglitazone (P1B), Sitagliptin (715), Linagliptin (356), Rosiglitazone (BRL), Vildagliptin (LF7) and Miglitol (MIG) may serve as diabetes drugs because ACR-IDE, P1B-IDE, 715-IDE, 356-IDE, BRL-IDE, and LF7-IDE are in H_0_, and MIG-IDE is in H_1_. Especially, we assert that Alogliptin (T22) and Saxagliptin (BJM) are better inhibitors of NEP other than the inhibitor of IDE because T22-IDE and BJM-IDE in H_2_, but T22-NEP in H_0_ and BJM-NEP in H_1_. Therefore, we may suggest alternatively using T22 and BJM as an inhibitor of NEP for heart failure rather than for diabetes.

Furthermore, P1B can stop the entire MMN of ACh Because of the facts, (1) P1B-ChAT is in H_0_, (2) P1B-CHRM2, P1B-CHRNA7, P1B-CHRNA9, P1B-CHRNB2 and P1B-CHRNE are in H_0_ or H_1_ and (3) P1B-AChE and P1B-BCHE are in H_0_. Similarly, we can show that P1B stops the entire MMN of Aβ. Since P1B crosses the BBB [39] and we can find the role of P1B to treat AD will be same as the role of E20, GNT, THA and HUP. Therefore, we suggest for these patients who have diabetes only, do not use P1B because it will weaken the learning and memory functions through restricting ACh binding to receptors.

Miglitol (MIG) only significantly inhibits AChE among all 49 proteins. Therefore it is possibly a good drug to improve the level of ACh, although it cannot cross BBB. In other words, MIG is not so effective for diabetes but it may be a potent AD drug.

### Implications of the data in Table E in S1 File

From Table E in S1 File, we may find that lisinopril (LPR), captopril (X8Z), and trandolapril (X93) are really good inhibitors of ACE because the dyadic arrays are (0.91, -7.7), (0.88, -8.8), and (0.91, -5.8), respectively. Moreover, X93 may lower the level of ACh since X93 crosses the BBB [40] and X93-ChAT falls into H_1_.

Telmisartan (TLS) is really an angiotensin II receptor blocker (ARB) because the dyadic array is (0.75, -8.4) and TLS may also be an inhibitor of ACE because the dyadic array is (0.77, -10.6). Moreover, the dyadic array of TLS-IDE is (0.74, -11.0) shows that TLS is also a good inhibitor of IDE. These computational results coincide with clinical experience that indicates that TLS is used as a diabetes drug and the effect of TLS is almost the same as the effect of lisinopril. Furthermore, TLS may paralyze both whole MMNs of ACh and Aβ and TLS crosses the BBB [41] imply that the role of TLS likes that of E20, GNT, THA, and HUP for AD. Therefore, TLS has potential multiple usages for HF, diabetes and AD, however it remains to be validated further.

Beta-blockers Atenolol (2TN) and Propranolol (SNP) can cross the BBB [42]. The dyadic array shows that 2TN is a safe drug because it only has the possibility to bind ACE, IDE and AChE. That is, 2TN may have an unexpected role to improve the level of ACh. While SNP has the possibility to inhibit ChAT, AChE and BCHE but does not inhibit all receptors on the MMN of ACh. Moreover, SNP may reduce Aβ production because SNP-BACE-1 is in H_1_. Therefore, SNP may prevent the Aβ deposition and may lower the level of ACh. Unlike E20, GNT, THA and HUP, SNP does not reject the infusion of ACh exogenously.

## Discussion and Conclusion

Overall, we generally define the MMN of a stimulus and use ACh and Aβ as the example to show the MMNs of ACh and Aβ. Then we show the network of AD generated by MMNs of ACh and Aβ, and the MMNs of other stimuli related to AD. Based on the network of AD, many new insights are emerged. For example, among the 30 drugs we selected to form the drug panel, we find that some inhibitors of a converting enzyme or a cleaning enzyme for some stimulus could paralyze the whole MMN of the stimulus, and even some blockers of the receptors also could paralyze the whole MMN of the stimulus. It will provide an insight to study the difference of the effects of a drug between *in vivo* and *in vitro* and an insight to analyze the drug resistance. Moreover, since enzymes often have multiple functions, an insight is emerged that some drugs cannot cure the appointed disease but probably induce a new disease.

In detail, we show some valued implications in this study to share with readers as below:

1. Despite the role of E20, GNT, THA and HUP is against to their original design for improving the level of ACh because it could paralyze the entire MMN of ACh and therefore the original level of ACh is unchanged because no producer and no consumers. However, they still benefit AD in the sense that E20, GNT, THA and HUP keep the Aβ deposition unchanged because it also could paralyze the whole MMN of Aβ. Therefore, we must note that E20, GNT, THA and HUP are useless if we want to use them to treat AD before Aβ deposition appearing. Especially, in this case the learning and memory ability will be harmed because ACh cannot be used by receptors, although we can infuse ACh to body exogenously.
2. The role 377 could not selectively block NMDAR but possible be the inhibitor of ChAT. Therefore, 377 is useless for AD, which seems against to the original design.
3. The role NAI and VIV for AD is similar to the role of E20, GNT, THA and HUP. NAI sounds stronger than E20, GNT, THA and HUP to stop the whole MMNs of Aβ and ACh, while VIV weaker than NAI. Therefore, NAI and VIV will be harmful if we want to use it to increase the level of ACh in preclinical AD. It suggests that nutrients also need to be controlled, due to their harmfulness if using in wrong time and wrong way.
4. Besides to treat hypertension or heart failure associated with diabetes, TLS has a new role related to AD. Since TLS crosses BBB and TLS could stop the entire MMN of Aβ, it follows that TLS keeps original level of Aβ deposition unchanged. This is good news for AD patients. TLS also stops the entire MMN of ACh. It also follows that original level of ACh is unchanged. However, ACh unbinding to receptors is a bad news for the learning and memory functions. Therefore, TLS is a safe drug for hypertension, heart failure, or diabetes, but could have a slight side effect to affect the learning and memory functions.
5. MIG hopefully is a potent drug to prevent the loss of ACh. Because it cannot cross BBB to interfere the proteins in brain and it just inhibits AChE which is a ubiquitous protein in central and peripheral nerve systems.
6. 2TN a safe drug for heart failure and hypertension since it can bind to ACE, but its role to treat heart failure and hypertension is minor. A new role to improve the level of ACh could be found in this study because it could only inhibit AChE on MMN of ACh. Therefore, 2TN is also a potent drug to prevent the loss of ACh.

More interesting conclusions could be mined from Tables C, D and E in S1 File, for example, both NAI and TLS are the basic elements of the source for discovering the multiple inhibitors in the natural way. Another example, amiloride is not only a diuretic but also a double inhibitor of IDE and NEP. Due to limited space, we only show these in this paper. Obviously, this study suggests that we should reconfirm the actual effects of each drug through using MMNs.

## Supporting Information

S1 File Supply materials for the network of AD: How to read the network of AD (Text A). The chemical names, functions and the 3D-structures of the 49 enzymes/receptors in the network of AD (Table A). The panel of 31 drugs consisted of 10 AD drugs, 9 diabetes drugs, and 12 heart failure drugs (Table B). The data for 490 complexes formed by 10 AD drugs and 49 proteins (Table C). The data for 441 complexes formed by 9 diabetes drugs and 49 proteins (Table D). The data for 539 complexes formed by 11 diabetes drugs and 49 proteins and the additional 49 complexes formed by the controlling drug amiloride (AMR) and 49 proteins (Table E). The simulation of IC50 data (Figs A-F).
(DOC)
