# Supplementary material for: Uncovering the Molecular Mechanism of Actions between Pharmaceuticals and Proteins on the AD Network
Source: PLoS One. 2015 Dec 9;10(12):e0144387. doi: 10.1371/journal.pone.0144387 (PMC4674063; doi:10.1371/journal.pone.0144387)
Supplement: S1 File — The chemical names, functions and the 3D-structures of the 49 enzymes/receptors in the network of AD (Table A). The panel of 31 drugs consisted of 10 AD drugs, 9 diabetes drugs, and 12 heart failure drugs (Table B). The data for 490 complexes formed by 10 AD drugs and 49 proteins (Table C). The data for 441 complexes formed by 9 diabetes drugs and 49 proteins (Table D). The data for 539 complexes formed by 11 diabetes drugs and 49 proteins and the additional 49 complexes formed by the controlling drug amiloride (AMR) and 49 proteins (Table E). The simulation of IC50 data (Figs A-F). (DOC) [file pone.0144387.s001.doc]

**Text A. How to read the network of AD**

**Figure 2** shows the network of Alzheimer's disease generated by the mini metabolic networks of beta amyloid, tau protein, acetylcholine, NF-kappaB and ARA. It is too complex to explain clearly within a small page. Therefore, we give the details of the network of AD here. There are three main symptoms including the senile plaques (SP) assumed as the abnormal accumulation of Aβ 42, the neurofibrillary tangles (NFT) assumed as the abnormal deposition of hyperphosphorylated tau proteins, and cognitive defect assumed as the lowered level of acetylcholine (ACh). We describe the connections among the mini metabolic networks in detail below.

**Where do Aβ** **and associated peptides come from?**

Aβ hypothesis assumes that deposition of Aβ 42 is the cause of SP which is one symptom of AD. The process converting amyloid precursor protein (APP) to Aβ needs two steps involving two proteolytic enzymes β-secretase (BACE-1) and γ-secretases with part of presenilin-1 (PS1) or presenilin-2 (PS2) [1]. This process is called as the amyloidogenic pathway. Typically, β-secretase (BACE-1) cleaves the luminal domain of APP, releasing sAPPβ and CTFβ, and then CTFβ of 99 residues is cleaved by γ-secretase to release Aβ 40/42 and CTFγ of 50 residues. As well as, APP can also be converted to other non-amyloid peptides by α-secretase competitively cleaves APP to release sAPPα and CTFα of 83 residues. And then CTFα is further cleaved by γ-secretase to release P3 of 24/26 residues and CTFγ [2-4].

**Which protein clean or decompose Aβ and associated peptides?**

Aβ is mainly cleaned by neutral endopeptidase (NEP) [5], endothelin converting enzyme (ECE)-1 [6], insulin degrading enzyme (IDE) [7] and angiotensin converting enzyme (ACE) [8]. The minor part is cleaned by cathepsin D [9], matrix metalloendopeptidase-2(MMP-2) [10], matrix metalloendopeptidase-9 (MMP-9) [11] and coagulation factor XIa [12]. The soluble APPsβ undergoes further cleavage to produce APP derivative (N-APP), acting via DR6 and caspase-6 with positive contributions to AD [13]. AICD has been shown to be further cleaved by caspases at amino acid 664 of APP (695 numbering) to release two smaller fragments, Jcasp and C31, the latter contains the last 31 amino acids of APP and has been proposed to mediate cytotoxicity in a full-length APP dependent manner [14-16].

**Receptors of Aβ**

The receptors of Aβ have been confirmed as NMDAR [17-19], Ephrin type B receptor 2 (EphB2) [20], Paired immunoglobulin-like receptor B (PirB) and Leukocyte immunoglobulin-like receptor B2 (LilrB2) [21], cellular prion protein (PrPC) [22-23], LRP1 [24], APOE [24-25], alpha7 AchR (CHRNA7), P75 neurotrophin receptor ( p75NTR), beta-adrenergic receptors (beta-ARS), and Fc γ receptor II-b (Fcγ RIIb) [26] . Especially, NMDAR is a unique receptor which has been used as the drug target. Moreover, the receptor LRP1 may also receive cleaning directly [24]. APOE can also be conceived of as a receptor of Aβ because it can combine with Aβ to form the complex of APOE and Aβ for cleaning Aβ in a new way [24-25].

**The interaction between NF-κB and Aβ**

NF-κB complex leads to up-regulates BACE1 expression and then up-regulate Aβ production from APP [27]. The NF-κB complex (p50-RelA heterodimers or p52-RelB heterodimers) are released through canonical and non-canonical pathways [28]. It follows that NF-κB may increase the activity of βAPP, BACE1 and γ–secretase so that Aβ concentrations become high [29]. Inversely, Aβ can activate NF-κB in cultured neurons [30-31] (highlighted with green arrow). By the two aspects, we may know the importance of the activity of NF-κB to form the senile plaque. Therefore how to suppress the activity of NF-κB may develop many potential therapies. For example, the intracellular ubiquitin-editing protein (A20) (also known as TNFAIP3) [32], the ubiquitin carboxyl-terminal hydrolase L1 (UCH-L1) [33], the ubiquitin carboxyl-terminal hydrolase (CYLD) [34], and the sirtuin type 1 (Sirt1) [35] are known as the enzymes with the capability of inhibit or attenuate the activity of NF-κB. How to activate them will get some self-cure therapies. Therefore, the mini pathway about NF-κB is added in the network of AD.

**The interaction between PLC and Aβ**

Phospholipase C (PLC) cleaves the phospholipid phosphatidylinositol 4,5-bisphosphate (PIP2) into diacyl glycerol (DAG) and inositol 1,4,5-trisphosphate (IP3) [36]. IP3 then diffuses through the cytosol to bind to IP3 receptors, particular calcium channels in the smooth endoplasmic reticulum (ER). In addition, Calcium enhances the proteolytic activity of BACE1 [37], but calcium and DAG together work to activate protein kinase C [38], which shift APP processing towards the non-amyloidogenic pathway [39].

**The interaction between AA and Aβ**

Arachidonic acid (AA) can be generated from [DAG](http://en.wikipedia.org/wiki/Diacylglycerol) by cytosolic PLA2 (cPLA2) [40]. The major pathways of AA metabolism are controlled by lipoxygenase (LOX), cyclooxygenase (COX), and cytochrome (CYP 1A1, 4A1, 4A2, and etc.) [41-42]. AA and its various metabolites (prostaglandins, thromboxanes, and leukotriene B4) resulted in significantly higher secretion of both Aβ 40 and 42 peptides [43]. Moreover, AA and its various metabolites (prostaglandin and leukotriene metabolites) may also activate NF-κB [44]. Therefore, how to lower the levels of AA and its various metabolites may also lower the activity of NF-κB and thus it is one of the ways to develop the self-cure therapies.

**The interaction between APOE, receptors of APOE and Aβ**

Prevailing data show that APOE genotypes strongly affect deposition of Aβ because that APOE and Aβ are co­deposited in senile plaques [45]. Especially, APOE4 is the strongest risk factor for late­onset AD [46]. Consistent studies show that insufficiency of APOE results in that amyloid plaque deposition is significantly decreased validated using amyloid mouse models [47]. Reducing the level of APOE might directly reduce Aβ deposition [48]. APOE4-lipoproteins bind Aβ with lower affinity than do APOE3-lipoproteins or APOE2-lipoproteins [49-50]. Several APOE receptors (i.e., LDLR-related protein 1 (LRP1), Low-density lipoprotein receptor (LDLR), VLDL receptor (VLDLR) and heparan sulfate proteoglycan (HSPG) is involved in receptor‑mediated clearance of Aβ when Aβ binds to APOE4-lipoproteins in the brain [24-25, 51 ]. In addition, LRP1 is likely to clear Aβ directly [24], LRP1 also has a role in facilitating APP processing to Aβ [24, 52].

**Tau and the related mini network**

Tau hypothesis assumes that tau proteins are abnormally phosphorylated and aggregates as paired helical filaments (PHFs) in neurofibrillary tangles (NFTs) [53–54]. Tau kinases involving glycogen synthase kinase-3β (GSK-3β) [55], microtubule afﬁnity regulating kinase (MARK) [56], cyclin-dependent kinase 5 (CDK5) [57] and casein kinase 1 (CK1) [57], plays a role in the formation of NFTs. It notes that PP2A is an enzyme to dephosphorylate [59].

**Acetylcholine (Ach) and the related mini network**

[Cholinergic](http://en.wikipedia.org/wiki/Cholinergic) hypothesis assumes that AD is caused by reduced synthesis of the [neurotransmitter](http://en.wikipedia.org/wiki/Neurotransmitter) acetylcholine (ACh) which is involved in both memory and learning. Choline acetyltransferase (ChAT) catalyzes the synthesis of ACh from choline and acetyl-coenzyme A in the presynaptic neuron and ACh is hydrolysed in the synaptic cleft by AChE tetramers [60] and butyrylcholinesterase (BChE) [61]. BChE can also act as a molecular decoy for natural anti-AChEs by reacting with these toxins before they reach AChE [62]. Muscarinic ACh receptor (mAChR) and nicotinic ACh receptor (nAChR) are the receptors of ACh, although the precise mechanism between AChRs and AD pathogenesis remains unclear. Stimulating the AChRs seems to be as a strategy for the treatment of AD [63].

**Crosstalk among Ach, Aβ and tau**

M1 mAChR and the M3 mAChR are predominantly found bound to G proteins of class Gq [64-65[]](http://en.wikipedia.org/wiki/Muscarinic_acetylcholine_receptor_M1#cite_note-pmid8645172-5), Gq work to activate [phospholipase C](http://en.wikipedia.org/wiki/Phosphoinositide_phospholipase_C) (PLC) [38], which involves activation of [protein kinase C](http://en.wikipedia.org/wiki/Protein_kinase_C), then regulate the α­secretase­mediated cleavage of APP [66-67]. So, Stimulation of the M1 mAChR and the M3 mAChR increases the PKC-mediated release of sAPP, implicating mAChR activation shift APP processing towards the non-amyloidogenic pathway and decrease Aβ production [68-69]. Aβ deposition may contribute to the cholinergic dysfunction in AD by decreasing the release of presynaptic ACh and impairing the coupling of postsynaptic M1 mAChRs with G proteins, which leads to decreas signal transduction, the more generation of neurotoxic Aβ and a further decrease in ACh release [70].

**M1 mAChR and Tau**

M1 mAChR activation reduces tau phosphorylation [71-72], muscarinic agonists reduce tau phosphorylation in non-neuronal cells via GSK-3β inhibition in neurons [72].

**α7 nAChRs and Aβ**

The α7 nAChRs mediate the neuron excitability, the neurotransmitter release and the induction of cognitive function [73-74]. Likewise, activation of this receptor improves attention, learning, and memory performance for the treatment of AD [75–76], Selective α7 AChRs agonists such as GTS-21 increase activate protein kinase C (PKC) [77], which shift APP processing towards the non-amyloidogenic pathway and decrease Aβ production. Aβ peptides and α7 nAChRs have a high-afﬁnity interaction, especially Aβ 42. Binding of Aβ 42 inhibits α7 nAChR-dependent calcium influx, which could explain the cognitive deficit of AD [78-79], and it suggests that α7nAChR is a key mediator of Aβ 42-induced tau protein phosphorylation [80]. Knocking out the a7nAChR in a transgenic mouse model of AD conferred a beneﬁt in learning and memory and preserved synaptic integrity compared to transgenic controls [81].

**α7nAChR inhibits NF-κB**

Nicotine suppresses the phosphorylation of I-κB, and then inhibits the transcriptional activity of NF-κB through α7nAChR. It may contribute to the regulation of some immune diseases [82]. The activated α7nAChR may rapidly inhibit the activity of IKK and therefore halt the phosphorylation of IκB and finally inhibit NF-κB.

**Aβ and Tau**

Aβ oligomers and Aβ ﬁbrils could induce tau phosphorylation [83-84], morphology changes of spines by missorting of endogenous tau into dendrites [85], and accelerate the formation of neuroﬁbrillary tangles (NFTs). Tau reduction or the absence of tau can block Aβ and excitotoxin induced neuronal dysfunction [86-87]. Tau targets the Src kinase Fyn to the dendritic compartment, and Fyn kinase phosphorylates the NMDA receptor (NMDAR) in dendritic spines, thereby mediating its interaction with the postsynaptic density protein 95 (PSD95), leading to enhance excitotoxicity. Excitotoxicity is known to increase the toxic effects of oligomers of Aβ on neurons [87].

**Table A. The chemical names, functions and the 3D-structures of the 49 enzymes/receptors in the network of AD**

| NO. | Enzyme/receptor | Function | Representatives of 3D-structure shown in PDB |
| --- | --- | --- | --- |
| 1 | A20 | An enzyme inhibits NF-κB | 3zje, 3zjd |
| 2 | ACE | The angiotensin converting enzyme, may also clean Aβ. | 1o86, 1o8a, 3nxq, 4aph (ACE); 4c2n, 3l3n, 4c2o (tACE) |
| 3 | AChE | An specific enzyme to hydrolyze acetylcholine | 1acj, 1eea, 1vot, 4bdt |
| 4 | ADAM10 | one of α-secretases | 2ao7 |
| 5 | ADAM17 | one of α-secretases | 2a8h; 1bkc, 1zxc (catalytic domain of TACE) |
| 6 | APOE2 | Weakly to promotes Aβ deposit | 1le2，1nfo (partial) |
| 7 | APOE3 | Weakly to promote Aβ deposit | 1bz4，1lpe，1nfn，2l7b （NMR） |
| 8 | APOE4 | the strongest risk factor to promote Aβ deposit | 1b68，1gs9，1le4 |
| 9 | BACE1 | β-secretase | 2hiz, 3uqp, 3uqw, 3uqx, 4jp9, 4jpc, 4jpe |
| 10 | BChE | as same as AChE for hydrolyzing acetylcholine | 2xmb, 2xmc, 4b0p |
| 11 | caspase-6 | N-APP acting via DR6 and caspase-6 | 2wdp,3k7e,3nkf |
| 12 | Cath-D | The cathepsin D, an enzyme may degrade Aβ | 1lya |
| 13 | CDK5 | A tau kinase may hyperphosphorylate tau | 1h4l, 1unl, 4au8 |
| 14 | ChAT | it catalyzes the synthesis of acetylcholine | 1t1u, 2fy2, 2fy3, 2fy4, 2fy5 |
| 15 | CHRM2 | M2 mAChR，muscarinic ACh receptors， | 3uon,4mqs,4mqt |
| 16 | CHRM3 | M3 mAChR，muscarinic ACh receptors | 2csa, 4daj |
| 17 | CHRNA1 | α1 nAChR， nicotinic ACh receptors | 1y5p |
| 18 | CHRNA4 | α4 nAChR， nicotinic ACh receptors | 2lly |
| 19 | CHRNA7 | α7 nAChR， nicotinic ACh receptors | 4hqp; 2maw (the alpha7 nAChR transmembrane domain) |
| 20 | CHRNA9 | α9 nAChR， nicotinic ACh receptors | 4uy2 |
| 21 | CHRNB2 | β2nAChR， nicotinic ACh receptors | 2ksr |
| 22 | CHRNB4 | β4nAChR， nicotinic ACh receptors | 2asg |
| 23 | CHRNE | nicotinic ACh receptors subunit epsilon | 2df9 |
| 24 | COX2 | It is involved in the metabolism of AA/ARA | 4cox (COX2) |
| 25 | CYLD | Protease, to inhibit the NF-κB | 1ixd,2vhf |
| 26 | CYP1A1 | involved in the metabolism of AA/ARA | 4i8v |
| 27 | DR6 | N-APP acting via DR6 and caspase-6 | 3qo4, 3u3p |
| 28 | ECE | endothelin converting enzyme, may also clean Aβ | 2pxx, 3dwb |
| 29 | EphB2 | One of the Aβ receptors | 1b4f,1nuk,3zfm |
| 30 | Factor XI | It may also degrade Aβ | 3bg8, 3sor |
| 31 | Fyn | it phosphorylates the NMDAR | 1aot_f, 1aou_f, 1g83 |
| 32 | GSK3β | tau kinases, to phosphorylate tau protein | 1gng,1h8f,1i09,1q3w,3q3b |
| 33 | IDE | Insulin degradation enzyme may also clean Aβ | 2g47,2g48,2g49,2jbu,2wk3,3qz2,4ifh, 4iof |
| 34 | IKK1, IKK2 | lead to release of the NF-κB | 3brt,3brv |
| 35 | InSP3R | IP3 receptors | 1n4k, 1xzz,3jrr |
| 36 | LilrB2 | One of the Aβ receptors | 2dyp_d, 4lla |
| 37 | LOX | lipoxygenase for the metabolism of AA/ARA | 2iuj,3o8y |
| 38 | MARK | tau kinases which may hyperphosphorylate tau | 1y8g,2r0i,2wzj,3iec |
| 39 | MM-2 | enzymes may also clean Aβ | 1ck7,1eak |
| 40 | MM-9 | enzymes may also clean Aβ | 1l6j,4h1q |
| 41 | NEMO | lead to release of the NF-κB | 3f89,3fx0 |
| 42 | NEP | neutral endopeptidase, it may also clean Aβ | 1r1h,1r1i,1r1j,2qpj,2yb9 |
| 43 | NMDARs | Aβ receptors | 3jpw,3nfl_e,3qel |
| 44 | PKC | An enzyme may reduce Aβ generation | 1gmi,1kpa,2fk9 |
| 45 | PLA2 | responsible for agonist-induced AA release | 2i0u,3g8g |
| 46 | PLC | PLC cleaves the PIP2 into DAG and IP3 | 1ah7 |
| 47 | PP2A | dephosphorylate tau | 2npp,2nyl,2nym,3dw8 |
| 48 | PrPC | One of the Aβ receptors | 4e1i,4e1i,4n9o |
| 49 | PS1 | Presenilin 1 | 2kr6 |

**Table B. The panel of 30 drugs consisted of 10 AD drugs, 9 diabetes drugs and 12 heart failure drugs**

| *No.* | *Drug name* | *drugbank ID* | *PDB name* | *target* | *function* |
| --- | --- | --- | --- | --- | --- |
| 1 | donepezil | DB00843 | E20 | AChE | AChE inhibitor |
| 2 | galantamine | DB00674 | GNT | AChE | AChE inhibitor |
| 3 | tacrine | DB00382 | THA | AChE | AChE inhibitor |
| 4 | Huperzine A | DB01928 | HUP | AChE | AChE inhibitor |
| 5 | memantine | DB01043 | 377 | NMDARs | a noncompetitive NMDAR antagonist |
| 6 | Choline | DB00122 | CHT | AChE | a partial precursor of acetylcholine |
| 7 | Phosphatidylserine | DB00144 | PSF | PKC-alpha | a phospholipid nutrient |
| 8 | Vitamin E | DB00163 | VIV/VIT | PKC-alpha | antioxidant activity |
| 9 | Lipoic Acid | DB00166 | LPB /LPA | LIPT1 | A vitamin-like antioxidant |
| 10 | NADH | DB00157 | NAI | UDPGDH | improving mental function |
| 11 | Rosiglitazone | DB00412 | BRL | PPARγ | Lowering the blood glucose |
| 12 | Pioglitazone | DB01132 | P1B | PPARγ | thiazolidinediones or "insulin sensitizers" |
| 13 | Linagliptin | [DB08882](http://www.drugbank.ca/drugs/DB08882) | 356 | DPP-4 | DPP-4 inhibitor |
| 14 | Vildagliptin | [DB04876](http://www.drugbank.ca/drugs/DB04876) | LF7 | DPP-4 | DPP-4 inhibitor |
| 15 | Alogliptin | DB06203 | T22 | DPP-4 | DPP-4 inhibitor |
| 16 | Sitagliptin | DB01261 | 715 | DPP-4 | DPP-4 inhibitor |
| 17 | Saxagliptin | DB06335 | BJM | DPP-4 | DPP-4 inhibitor |
| 18 | Miglitol | DB00491 | MIG | MGAM | alpha glucosidase inhibitor |
| 19 | Acarbose | DB00284 | ACR/QPS | MGAM | alpha glucosidase inhibitor |
| 20 | Lisinopril | DB00722 | LPR | ACE | ACE inhibitor |
| 21 | captopril | DB01197 | X8Z | ACE | ACE inhibitor |
| 22 | Trandolapril | DB00519 | X93 | ACE | ACE inhibitor |
| 23 | Telmisartan | DB00966 | TLS | AT1 or PPARγ | angiotensin II receptor blocker (ARB) |
| 24 | Clonidine | DB00575 | CLU | Alpha-2AAR | alpha agonist |
| 25 | Furosemide | DB00695 | FUN | CA2 | diuretic |
| 26 | Carvedilol | DB01136 | CVD | ADRB1 | alpha- beta-blocker |
| 27 | Timolol | DB00373 | TIM | ADRB1 | beta-blocker |
| 28 | Atenolol | DB00335 | 2TN | ADRB1 | beta-blocker |
| 29 | Propranolol | DB00571 | SNP | ADRB1 | beta-blocker |
| 30 | Amlodipine | DB00381 | 06X | CA1 | calcium channel blocker |
| 31 | Amiloride | DB00594 | AMR | ASIC1 | inhibiting sodium reabsorption |

**Table C.** **The values of similarity and free energy for 490 complexes formed by 10 AD drugs and 49 proteins**

| **name** | **E20** | | **GNT** | | **THA** | | **HUP** | | **377** | | **LPA** | | **CHT** | | **PSF** | | **VIV** | | **NAI** | |
| --- | --- | --- | --- | --- | --- | --- | --- | --- | --- | --- | --- | --- | --- | --- | --- | --- | --- | --- | --- | --- |
|  | *x*_1_ | *x*_2_ | *x*_1_ | *x*_2_ | *x*_1_ | *x*_2_ | *x*_1_ | *x*_2_ | *x*_1_ | *x*_2_ | *x*_1_ | *x*_2_ | *x*_1_ | *x*_2_ | *x*_1_ | *x*_2_ | *x*_1_ | *x*_2_ | *x*_1_ | *x*_2_ |
| A20 | 0.67 | -7.0 | 0.65 | -7.2 | 0.65 | -6.7 | 0.66 | -7.3 | 0.50 | -6.0 | 0.56 | -4.8 | 0.41 | -3.5 | 0.41 | -5.8 | 0.71 | -6.6 | 0.78 | -7.4 |
| ACE | 0.75 | -9.0 | 0.75 | -8.0 | 0.74 | -7.1 | 0.76 | -8.5 | 0.56 | -7.1 | 0.61 | -5.4 | 0.45 | -3.8 | 0.46 | -6.6 | 0.73 | -7.7 | 0.85 | -10.1 |
| AChE | 0.90 | -8.2 | 0.90 | -8.8 | 0.90 | -8.0 | 0.90 | -9.6 | 0.51 | -7.8 | 0.65 | -5.8 | 0.72 | -3.7 | 0.44 | -5.6 | 0.69 | -8.1 | 0.82 | -9.3 |
| ADAM10 | 0.45 | -6.5 | 0.47 | -6.9 | 0.48 | -5.7 | 0.47 | -6.7 | 0.44 | -4.8 | 0.46 | -4.4 | 0.39 | -3.0 | 0.47 | -5.7 | 0.44 | -6.0 | 0.57 | -7.3 |
| ADAM17 | 0.53 | -6.9 | 0.54 | -7.2 | 0.53 | -7.1 | 0.55 | -7.3 | 0.46 | -6.1 | 0.54 | -5.2 | 0.41 | -3.5 | 0.49 | -6.0 | 0.56 | -6.8 | 0.70 | -8.2 |
| APOE2 | 0.46 | -7.1 | 0.46 | -6.4 | 0.49 | -6.4 | 0.45 | -6.8 | 0.51 | -6.2 | 0.50 | -4.4 | 0.39 | -3.2 | 0.50 | -5.0 | 0.57 | -6.6 | 0.63 | -7.3 |
| APOE3 | 0.42 | -7.3 | 0.43 | -6.9 | 0.43 | -6.4 | 0.42 | -7.0 | 0.43 | -5.8 | 0.43 | -4.5 | 0.38 | -3.2 | 0.43 | -5.1 | 0.46 | -6.5 | 0.53 | -6.9 |
| APOE4 | 0.43 | -6.6 | 0.45 | -6.3 | 0.45 | -5.9 | 0.43 | -6.2 | 0.48 | -5.0 | 0.45 | -4.2 | 0.38 | -2.9 | 0.48 | -5.0 | 0.49 | -6.1 | 0.57 | -6.3 |
| BACE1 | 0.67 | -7.6 | 0.70 | -7.2 | 0.69 | -7.1 | 0.70 | -7.2 | 0.50 | -6.1 | 0.64 | -5.2 | 0.42 | -3.4 | 0.40 | -6.1 | 0.69 | -7.3 | 0.82 | -8.9 |
| BCHE | 0.90 | -8.4 | 0.90 | -8.4 | 0.89 | -7.7 | 0.90 | -8.1 | 0.51 | -6.5 | 0.65 | -5.0 | 0.68 | -3.5 | 0.51 | -6.1 | 0.67 | -8.2 | 0.82 | -9.0 |
| casp-6 | 0.58 | -8.5 | 0.58 | -7.2 | 0.63 | -7.2 | 0.60 | -7.5 | 0.46 | -6.5 | 0.52 | -5.2 | 0.43 | -3.7 | 0.49 | -5.6 | 0.70 | -7.3 | 0.72 | -8.7 |
| Cath- D | 0.51 | -7.6 | 0.50 | -7.7 | 0.48 | -6.9 | 0.46 | -7.9 | 0.44 | -6.0 | 0.50 | -5.4 | 0.43 | -3.4 | 0.52 | -5.8 | 0.59 | -6.1 | 0.61 | -8.4 |
| CDK5 | 0.60 | -7.3 | 0.61 | -7.5 | 0.58 | -7.3 | 0.59 | -6.6 | 0.51 | -6.1 | 0.57 | -5.3 | 0.41 | -3.7 | 0.45 | -6.1 | 0.68 | -7.5 | 0.74 | -8.7 |
| ChAT | 0.67 | -7.5 | 0.67 | -7.4 | 0.70 | -6.7 | 0.72 | -7.5 | 0.55 | -6.5 | 0.61 | -5.0 | 0.67 | -3.6 | 0.51 | -6.0 | 0.69 | -7.2 | 0.83 | -8.5 |
| CHRM2 | 0.64 | -8.1 | 0.67 | -9.2 | 0.64 | -7.7 | 0.62 | -7.5 | 0.52 | -7.0 | 0.59 | -5.6 | 0.46 | -3.6 | 0.48 | -6.6 | 0.73 | -8.1 | 0.79 | -9.6 |
| CHRM3 | 0.53 | -6.5 | 0.52 | -7.4 | 0.53 | -5.9 | 0.50 | -7.6 | 0.43 | -6.1 | 0.45 | -4.7 | 0.39 | -3.3 | 0.42 | -5.1 | 0.52 | -6.3 | 0.58 | -7.8 |
| CHRNA1 | 0.31 | -6.2 | 0.31 | -5.7 | 0.32 | -5.5 | 0.31 | -5.6 | 0.31 | -4.8 | 0.31 | -4.4 | 0.31 | -3.2 | 0.31 | -4.8 | 0.32 | -6.2 | 0.32 | -6.7 |
| CHRNA4 | 0.58 | -9.4 | 0.61 | -7.9 | 0.61 | -7.1 | 0.60 | -8.0 | 0.45 | -7.1 | 0.52 | -5.3 | 0.33 | -3.4 | 0.40 | -6.2 | 0.46 | -8.2 | 0.68 | -8.5 |
| CHRNA7 | 0.51 | -7.9 | 0.55 | -7.0 | 0.57 | -7.2 | 0.54 | -8.1 | 0.49 | -6.6 | 0.52 | -5.0 | 0.36 | -3.5 | 0.42 | -5.4 | 0.56 | -7.0 | 0.67 | -8.6 |
| CHRNA9 | 0.53 | -7.4 | 0.58 | -8.5 | 0.51 | -8.0 | 0.51 | -7.7 | 0.52 | -6.4 | 0.47 | -5.4 | 0.40 | -3.5 | 0.47 | -6.1 | 0.60 | -7.7 | 0.64 | -8.7 |
| CHRNB2 | 0.58 | -6.9 | 0.59 | -6.4 | 0.61 | -6.5 | 0.60 | -7.3 | 0.47 | -6.1 | 0.50 | -5.3 | 0.33 | -3.2 | 0.41 | -6.0 | 0.49 | -7.6 | 0.72 | -7.5 |
| CHRNB4 | 0.31 | -9.0 | 0.31 | -7.3 | 0.31 | -7.0 | 0.31 | -7.7 | 0.32 | -6.2 | 0.32 | -4.9 | 0.31 | -3.0 | 0.32 | -5.7 | 0.32 | -8.2 | 0.32 | -7.8 |
| CHRNE | 0.49 | -8.1 | 0.51 | -7.1 | 0.56 | -6.9 | 0.46 | -8.0 | 0.46 | -6.3 | 0.48 | -4.7 | 0.46 | -2.9 | 0.42 | -5.8 | 0.59 | -8.4 | 0.60 | -7.7 |
| COX | 0.70 | -9.1 | 0.74 | -8.2 | 0.74 | -7.8 | 0.68 | -8.0 | 0.52 | -6.5 | 0.59 | -6.0 | 0.42 | -3.7 | 0.36 | -7.3 | 0.72 | -7.6 | 0.83 | -11.6 |
| CYLD | 0.64 | -7.4 | 0.64 | -6.9 | 0.63 | -6.3 | 0.62 | -6.6 | 0.47 | -6.2 | 0.62 | -4.5 | 0.40 | -3.3 | 0.48 | -5.4 | 0.61 | -6.1 | 0.78 | -8.3 |
| CYP1A1 | 0.62 | -7.3 | 0.67 | -6.9 | 0.68 | -6.5 | 0.67 | -7.1 | 0.55 | -6.4 | 0.58 | -4.4 | 0.40 | -3.6 | 0.32 | -5.9 | 0.67 | -6.3 | 0.80 | -8.8 |
| DR6 | 0.42 | -6.2 | 0.46 | -5.8 | 0.44 | -5.4 | 0.41 | -5.8 | 0.43 | -5.0 | 0.42 | -4.0 | 0.38 | -3.0 | 0.42 | -4.7 | 0.44 | -5.1 | 0.51 | -6.4 |
| ECE | 0.67 | -9.2 | 0.68 | -7.9 | 0.68 | -7.0 | 0.70 | -8.2 | 0.53 | -6.7 | 0.65 | -6.0 | 0.44 | -3.8 | 0.47 | -6.2 | 0.66 | -8.5 | 0.82 | -8.7 |
| EphB2 | 0.50 | -6.9 | 0.51 | -6.7 | 0.50 | -6.1 | 0.50 | -6.9 | 0.46 | -5.6 | 0.49 | -4.6 | 0.41 | -3.2 | 0.42 | -5.6 | 0.55 | -6.5 | 0.65 | -7.3 |
| Factor X1a | 0.53 | -7.5 | 0.56 | -7.6 | 0.53 | -7.1 | 0.58 | -7.1 | 0.46 | -5.4 | 0.51 | -4.9 | 0.38 | -3.6 | 0.48 | -6.1 | 0.54 | -6.0 | 0.68 | -9.1 |
| Fyn | 0.50 | -7.2 | 0.51 | -6.8 | 0.49 | -6.0 | 0.52 | -6.8 | 0.44 | -5.8 | 0.49 | -4.6 | 0.38 | -3.5 | 0.43 | -5.3 | 0.50 | -5.9 | 0.63 | -7.2 |
| GSK3β | 0.60 | -7.9 | 0.62 | -7.6 | 0.65 | -7.1 | 0.61 | -7.4 | 0.55 | -6.5 | 0.62 | -4.9 | 0.43 | -3.4 | 0.47 | -6.5 | 0.71 | -7.6 | 0.78 | -9.2 |
| IDE | 0.75 | -8.3 | 0.75 | -8.1 | 0.74 | -8.7 | 0.77 | -8.2 | 0.60 | -6.7 | 0.66 | -5.9 | 0.49 | -3.6 | 0.50 | -6.4 | 0.72 | -8.0 | 0.85 | -8.9 |
| IKK1,IKK2 | 0.32 | -6.1 | 0.31 | -5.8 | 0.32 | -6.2 | 0.32 | -5.8 | 0.33 | -5.0 | 0.32 | -4.4 | 0.31 | -3.5 | 0.46 | -5.0 | 0.31 | -6.7 | 0.33 | -7.2 |
| InSP3R | 0.54 | -7.2 | 0.53 | -7.0 | 0.54 | -6.4 | 0.55 | -6.6 | 0.49 | -5.6 | 0.53 | -4.8 | 0.42 | -3.1 | 0.49 | -5.5 | 0.65 | -6.9 | 0.74 | -8.4 |
| LilrB2 | 0.49 | -7.9 | 0.48 | -7.5 | 0.51 | -6.9 | 0.47 | -7.9 | 0.47 | -6.3 | 0.55 | -4.8 | 0.39 | -3.4 | 0.45 | -6.1 | 0.58 | -7.6 | 0.66 | -9.2 |
| LOX | 0.71 | -8.4 | 0.73 | -7.4 | 0.73 | -7.9 | 0.70 | -8.1 | 0.60 | -6.7 | 0.65 | -5.4 | 0.42 | -3.7 | 0.49 | -5.9 | 0.71 | -7.1 | 0.82 | -9.1 |
| MARK, | 0.61 | -7.9 | 0.61 | -7.8 | 0.62 | -7.1 | 0.58 | -7.7 | 0.51 | -6.8 | 0.58 | -5.2 | 0.44 | -3.4 | 0.47 | -6.3 | 0.70 | -7.8 | 0.78 | -8.7 |
| MM-2 | 0.65 | -7.9 | 0.67 | -7.8 | 0.69 | -7.4 | 0.65 | -8.0 | 0.55 | -7.1 | 0.66 | -5.7 | 0.44 | -3.8 | 0.50 | -6.1 | 0.69 | -7.3 | 0.83 | -8.8 |
| MM-9 | 0.54 | -8.8 | 0.54 | -7.7 | 0.55 | -8.3 | 0.56 | -7.9 | 0.47 | -6.7 | 0.56 | -5.5 | 0.41 | -3.8 | 0.41 | -6.5 | 0.57 | -7.5 | 0.71 | -9.4 |
| NEMO | 0.31 | -5.5 | 0.31 | -5.7 | 0.33 | -4.9 | 0.31 | -5.3 | 0.34 | -4.6 | 0.33 | -3.7 | 0.31 | -2.5 | 0.49 | -4.2 | 0.31 | -4.9 | 0.34 | -5.5 |
| NEP | 0.69 | -8.0 | 0.73 | -7.5 | 0.72 | -6.7 | 0.73 | -7.3 | 0.55 | -6.4 | 0.66 | -5.2 | 0.44 | -3.9 | 0.49 | -5.9 | 0.67 | -7.3 | 0.85 | -9.3 |
| NMDAR | 0.49 | -7.0 | 0.51 | -7.2 | 0.50 | -6.8 | 0.52 | -7.2 | 0.43 | -5.9 | 0.47 | -5.0 | 0.37 | -3.4 | 0.40 | -5.5 | 0.52 | -6.3 | 0.62 | -7.5 |
| PKC | 0.45 | -6.9 | 0.45 | -6.2 | 0.52 | -6.1 | 0.40 | -6.4 | 0.44 | -5.3 | 0.45 | -4.4 | 0.41 | -3.0 | 0.51 | -5.3 | 0.49 | -6.7 | 0.59 | -7.5 |
| PLA2 | 0.51 | -8.1 | 0.49 | -7.4 | 0.49 | -7.1 | 0.47 | -8.0 | 0.41 | -6.4 | 0.49 | -5.1 | 0.40 | -4.0 | 0.53 | -5.9 | 0.56 | -7.4 | 0.63 | -8.1 |
| PLC | 0.54 | -8.1 | 0.55 | -7.9 | 0.60 | -7.4 | 0.60 | -8.1 | 0.47 | -6.2 | 0.52 | -6.2 | 0.41 | -3.9 | 0.50 | -6.0 | 0.61 | -6.9 | 0.71 | -8.1 |
| PP2A | 0.62 | -7.2 | 0.62 | -7.7 | 0.65 | -6.8 | 0.63 | -7.5 | 0.53 | -6.5 | 0.55 | -5.3 | 0.42 | -3.7 | 0.44 | -6.2 | 0.61 | -6.8 | 0.82 | -8.9 |
| PrPC | 0.38 | -5.7 | 0.39 | -5.8 | 0.41 | -5.5 | 0.38 | -6.1 | 0.38 | -5.1 | 0.40 | -4.1 | 0.36 | -3.2 | 0.39 | -4.8 | 0.42 | -5.1 | 0.48 | -7.0 |
| PS1 | 0.44 | -7.1 | 0.40 | -6.5 | 0.51 | -6.6 | 0.44 | -6.8 | 0.42 | -6.0 | 0.44 | -5.0 | 0.38 | -3.5 | 0.56 | -5.4 | 0.56 | -6.9 | 0.57 | -7.1 |

**Table D. The values of similarity and free energy for 441 complexes formed by 9 diabetes drugs and 49** proteins

| **name** | **ACR** | | **P1B** | | **BRL** | | **715** | | **356** | | **LF7** | | **MIG** | | **T22** | | **BJM** | |
| --- | --- | --- | --- | --- | --- | --- | --- | --- | --- | --- | --- | --- | --- | --- | --- | --- | --- | --- |
|  | *x*_1_ | *x*_2_ | *x*_1_ | *x*_2_ | *x*_1_ | *x*_2_ | *x*_1_ | *x*_2_ | *x*_1_ | *x*_2_ | *x*_1_ | *x*_2_ | *x*_1_ | *x*_2_ | *x*_1_ | *x*_2_ | *x*_1_ | *x*_2_ |
| A20 | 0.65 | -7.5 | 0.70 | -7.1 | 0.67 | -6.7 | 0.56 | -7.8 | 0.38 | -7.9 | 0.52 | -7.2 | 0.49 | -5.2 | 0.47 | -7.1 | 0.43 | -6.9 |
| ACE | 0.77 | -8.7 | 0.78 | -8.5 | 0.77 | -8.0 | 0.67 | -9.3 | 0.47 | -9.8 | 0.63 | -8.2 | 0.70 | -6.0 | 0.46 | -8.1 | 0.40 | -8.1 |
| AChE | 0.75 | -8.0 | 0.74 | -8.3 | 0.70 | -7.1 | 0.65 | -8.7 | 0.49 | -8.6 | 0.67 | -7.5 | 0.69 | -6.0 | 0.41 | -7.5 | 0.37 | -7.7 |
| ADAM10 | 0.48 | -6.1 | 0.50 | -6.3 | 0.50 | -6.1 | 0.38 | -7.8 | 0.43 | -7.6 | 0.42 | -5.8 | 0.42 | -4.8 | 0.35 | -5.7 | 0.34 | -6.0 |
| ADAM17 | 0.60 | -7.4 | 0.60 | -7.4 | 0.57 | -6.9 | 0.51 | -8.3 | 0.37 | -8.0 | 0.48 | -6.9 | 0.53 | -5.5 | 0.36 | -6.6 | 0.35 | -7.1 |
| APOE2 | 0.50 | -6.2 | 0.56 | -6.7 | 0.55 | -6.6 | 0.43 | -6.6 | 0.35 | -7.4 | 0.39 | -6.3 | 0.38 | -5.0 | 0.35 | -6.4 | 0.34 | -6.2 |
| APOE3 | 0.47 | -6.4 | 0.49 | -6.8 | 0.50 | -6.4 | 0.42 | -7.1 | 0.34 | -7.5 | 0.38 | -6.5 | 0.39 | -4.7 | 0.34 | -6.8 | 0.34 | -6.2 |
| APOE4 | 0.48 | -5.9 | 0.52 | -6.3 | 0.55 | -6.2 | 0.45 | -6.6 | 0.35 | -6.8 | 0.39 | -5.9 | 0.41 | -4.4 | 0.34 | -5.9 | 0.34 | -6.1 |
| BACE1 | 0.74 | -7.5 | 0.72 | -8.1 | 0.69 | -7.1 | 0.60 | -8.6 | 0.39 | -8.8 | 0.56 | -7.5 | 0.65 | -5.5 | 0.39 | -6.9 | 0.37 | -7.0 |
| BCHE | 0.73 | -8.7 | 0.72 | -8.3 | 0.70 | -7.9 | 0.62 | -9.0 | 0.44 | -9.9 | 0.58 | -7.6 | 0.68 | -5.5 | 0.40 | -8.1 | 0.37 | -7.7 |
| casp-6 | 0.62 | -7.8 | 0.66 | -8.3 | 0.67 | -7.3 | 0.51 | -8.5 | 0.37 | -8.6 | 0.50 | -7.2 | 0.53 | -5.7 | 0.37 | -7.3 | 0.36 | -7.1 |
| Cath- D | 0.52 | -7.5 | 0.68 | -7.5 | 0.52 | -6.8 | 0.43 | -8.3 | 0.44 | -8.5 | 0.39 | -7.0 | 0.36 | -5.5 | 0.37 | -6.7 | 0.33 | -7.3 |
| CDK5 | 0.66 | -6.8 | 0.66 | -7.6 | 0.68 | -7.2 | 0.52 | -8.3 | 0.37 | -8.2 | 0.50 | -6.8 | 0.54 | -5.6 | 0.39 | -7.0 | 0.36 | -6.7 |
| ChAT | 0.76 | -7.6 | 0.74 | -7.6 | 0.68 | -7.8 | 0.66 | -8.4 | 0.40 | -8.7 | 0.61 | -7.9 | 0.70 | -5.6 | 0.40 | -7.1 | 0.42 | -7.4 |
| CHRM2 | 0.68 | -7.7 | 0.73 | -8.0 | 0.72 | -8.2 | 0.59 | -9.2 | 0.47 | -8.6 | 0.53 | -8.0 | 0.54 | -5.9 | 0.40 | -7.3 | 0.38 | -7.5 |
| CHRM3 | 0.55 | -6.6 | 0.54 | -7.1 | 0.51 | -6.6 | 0.47 | -7.4 | 0.35 | -7.7 | 0.46 | -6.9 | 0.45 | -4.9 | 0.35 | -6.5 | 0.34 | -7.0 |
| CHRNA1 | 0.32 | -6.1 | 0.31 | -6.1 | 0.32 | -5.2 | 0.32 | -6.8 | 0.31 | -7.7 | 0.32 | -5.9 | 0.31 | -4.1 | 0.31 | -6.0 | 0.31 | -5.4 |
| CHRNA4 | 0.62 | -6.2 | 0.53 | -8.6 | 0.49 | -8.5 | 0.55 | -10.2 | 0.31 | -8.6 | 0.54 | -8.4 | 0.56 | -5.2 | 0.31 | -8.8 | 0.31 | -8.5 |
| CHRNA7 | 0.58 | -7.2 | 0.61 | -8.0 | 0.54 | -7.1 | 0.52 | -7.7 | 0.34 | -8.6 | 0.50 | -6.6 | 0.51 | -5.3 | 0.34 | -6.9 | 0.33 | -7.1 |
| CHRNA9 | 0.57 | -7.7 | 0.58 | -9.3 | 0.60 | -8.2 | 0.44 | -9.2 | 0.35 | -7.8 | 0.43 | -8.1 | 0.44 | -5.5 | 0.35 | -8.1 | 0.35 | -6.7 |
| CHRNB2 | 0.65 | -5.2 | 0.61 | -7.7 | 0.54 | -7.3 | 0.58 | -8.5 | 0.31 | -7.6 | 0.55 | -6.2 | 0.60 | -4.9 | 0.31 | -6.5 | 0.31 | -6.5 |
| CHRNB4 | 0.32 | -7.9 | 0.32 | -8.7 | 0.32 | -8.2 | 0.31 | -9.1 | 0.31 | -10.4 | 0.31 | -7.2 | 0.31 | -4.7 | 0.31 | -7.8 | 0.31 | -7.4 |
| CHRNE | 0.50 | -5.9 | 0.63 | -7.9 | 0.64 | -6.7 | 0.40 | -8.1 | 0.34 | -8.7 | 0.37 | -7.5 | 0.38 | -4.4 | 0.34 | -8.0 | 0.33 | -7.8 |
| COX | 0.75 | -10.0 | 0.76 | -9.0 | 0.77 | -9.0 | 0.63 | -9.8 | 0.40 | -10.7 | 0.59 | -8.8 | 0.66 | -6.3 | 0.67 | -8.3 | 0.36 | -8.0 |
| CYLD | 0.57 | -6.9 | 0.62 | -7.1 | 0.65 | -6.4 | 0.50 | -7.1 | 0.38 | -8.4 | 0.47 | -6.7 | 0.49 | -5.1 | 0.39 | -6.3 | 0.35 | -6.6 |
| CYP1A1 | 0.70 | -7.7 | 0.73 | -9.9 | 0.71 | -6.5 | 0.62 | -9.5 | 0.38 | -9.2 | 0.57 | -7.9 | 0.66 | -5.6 | 0.38 | -6.8 | 0.37 | -7.9 |
| DR6 | 0.48 | -6.1 | 0.45 | -6.2 | 0.45 | -5.8 | 0.38 | -6.7 | 0.37 | -6.7 | 0.33 | -5.9 | 0.43 | -4.9 | 0.37 | -5.6 | 0.33 | -5.6 |
| ECE | 0.70 | -7.5 | 0.71 | -8.6 | 0.67 | -9.1 | 0.62 | -9.1 | 0.53 | -9.2 | 0.57 | -8.0 | 0.64 | -5.9 | 0.55 | -7.5 | 0.52 | -7.7 |
| EphB2 | 0.52 | -6.3 | 0.64 | -6.7 | 0.59 | -6.3 | 0.45 | -7.5 | 0.36 | -7.9 | 0.40 | -6.6 | 0.43 | -5.1 | 0.41 | -6.5 | 0.35 | -6.2 |
| Factor XI | 0.57 | -7.3 | 0.56 | -7.3 | 0.42 | -7.4 | 0.48 | -8.4 | 0.37 | -8.5 | 0.47 | -7.2 | 0.47 | -6.1 | 0.38 | -7.0 | 0.35 | -6.9 |
| Fyn | 0.55 | -6.7 | 0.52 | -6.8 | 0.50 | -6.7 | 0.48 | -7.4 | 0.34 | -7.3 | 0.44 | -6.5 | 0.46 | -5.2 | 0.36 | -6.4 | 0.33 | -6.4 |
| GSK3β | 0.69 | -8.0 | 0.70 | -8.2 | 0.68 | -7.4 | 0.58 | -8.6 | 0.51 | -8.9 | 0.57 | -7.6 | 0.59 | -5.4 | 0.53 | -7.1 | 0.43 | -7.8 |
| IDE | 0.81 | -8.2 | 0.79 | -8.6 | 0.78 | -8.2 | 0.77 | -9.6 | 0.77 | -9.4 | 0.75 | -8.4 | 0.73 | -5.9 | 0.47 | -7.8 | 0.43 | -7.7 |
| IKK1,IKK2 | 0.33 | -6.5 | 0.33 | -6.1 | 0.33 | -6.0 | 0.31 | -6.9 | 0.31 | -7.0 | 0.31 | -6.0 | 0.31 | -4.5 | 0.31 | -5.7 | 0.31 | -6.2 |
| InSP3R | 0.64 | -7.4 | 0.65 | -6.9 | 0.65 | -6.7 | 0.53 | -7.4 | 0.42 | -7.9 | 0.48 | -7.0 | 0.52 | -5.1 | 0.38 | -6.4 | 0.36 | -7.1 |
| LilrB2 | 0.56 | -8.2 | 0.59 | -8.2 | 0.55 | -7.1 | 0.47 | -8.9 | 0.39 | -9.4 | 0.44 | -7.7 | 0.47 | -5.8 | 0.41 | -7.1 | 0.36 | -8.0 |
| LOX | 0.78 | -8.2 | 0.76 | -8.1 | 0.73 | -7.1 | 0.71 | -8.9 | 0.72 | -9.4 | 0.68 | -7.5 | 0.74 | -6.2 | 0.57 | -7.8 | 0.38 | -7.8 |
| MARK, | 0.67 | -7.8 | 0.71 | -7.6 | 0.66 | -7.2 | 0.57 | -8.6 | 0.44 | -9.1 | 0.51 | -7.6 | 0.58 | -5.4 | 0.50 | -7.7 | 0.36 | -7.5 |
| MM-2 | 0.76 | -8.6 | 0.72 | -8.2 | 0.71 | -7.1 | 0.66 | -8.8 | 0.40 | -9.5 | 0.62 | -7.7 | 0.68 | -6.0 | 0.52 | -7.6 | 0.37 | -7.8 |
| MM-9 | 0.64 | -8.1 | 0.62 | -8.6 | 0.58 | -9.1 | 0.52 | -9.1 | 0.49 | -9.3 | 0.50 | -7.5 | 0.53 | -5.7 | 0.38 | -7.7 | 0.35 | -7.8 |
| NEMO | 0.34 | -5.3 | 0.33 | -5.6 | 0.33 | -5.6 | 0.32 | -6.1 | 0.31 | -6.1 | 0.31 | -5.3 | 0.31 | -3.9 | 0.32 | -4.9 | 0.31 | -5.4 |
| NEP | 0.79 | -8.3 | 0.83 | -7.9 | 0.72 | -7.7 | 0.71 | -8.8 | 0.39 | -9.1 | 0.69 | -8.1 | 0.71 | -5.8 | 0.70 | -7.2 | 0.66 | -7.9 |
| NMDAR | 0.55 | -7.3 | 0.56 | -7.0 | 0.49 | -6.7 | 0.49 | -7.4 | 0.35 | -7.8 | 0.47 | -7.0 | 0.51 | -5.5 | 0.36 | -6.7 | 0.34 | -7.2 |
| PKC | 0.48 | -6.7 | 0.52 | -6.7 | 0.47 | -6.7 | 0.43 | -7.4 | 0.36 | -7.5 | 0.40 | -6.6 | 0.38 | -4.9 | 0.35 | -6.0 | 0.34 | -6.0 |
| PLA2 | 0.54 | -7.5 | 0.56 | -7.8 | 0.57 | -7.1 | 0.42 | -8.6 | 0.36 | -8.8 | 0.42 | -7.3 | 0.46 | -6.3 | 0.40 | -7.4 | 0.35 | -7.7 |
| PLC | 0.63 | -7.4 | 0.65 | -8.4 | 0.55 | -8.0 | 0.55 | -8.8 | 0.40 | -8.1 | 0.55 | -7.5 | 0.60 | -5.6 | 0.42 | -7.9 | 0.36 | -7.2 |
| PP2A | 0.70 | -8.7 | 0.67 | -7.6 | 0.65 | -7.3 | 0.61 | -8.3 | 0.41 | -9.2 | 0.59 | -7.3 | 0.63 | -6.0 | 0.38 | -7.7 | 0.35 | -7.8 |
| PrPC | 0.40 | -6.2 | 0.42 | -5.9 | 0.44 | -5.6 | 0.38 | -6.9 | 0.33 | -6.8 | 0.36 | -6.3 | 0.37 | -4.8 | 0.34 | -5.8 | 0.33 | -6.3 |
| PS1 | 0.44 | -6.4 | 0.52 | -6.8 | 0.43 | -6.6 | 0.37 | -6.8 | 0.39 | -7.9 | 0.35 | -6.0 | 0.36 | -4.7 | 0.36 | -7.1 | 0.34 | -5.6 |

**Table E. The values of similarity and free energy for 539 complexes formed by 11 HF drugs and 49 proteins and the additional 49 complexes formed by the controlling drug AMR and 49 proteins.**

| **name** | **LPR** | | **X93** | | **X8Z** | | **TLS** | | **CLU** | | **CVD** | | **TIM** | | **FUN** | | **06X** | | **SNP** | | **2TN** | | **AMR** | |
| --- | --- | --- | --- | --- | --- | --- | --- | --- | --- | --- | --- | --- | --- | --- | --- | --- | --- | --- | --- | --- | --- | --- | --- | --- |
|  | *x*_1_ | *x*_2_ | *x*_1_ | *x*_2_ | *x*_1_ | *x*_2_ | *x*_1_ | *x*_2_ | *x*_1_ | *x*_2_ | *x*_1_ | *x*_2_ | *x*_1_ | *x*_2_ | *x*_1_ | *x*_2_ | *x*_1_ | *x*_2_ | *x*_1_ | *x*_2_ | *x*_1_ | *x*_2_ | *x*_1_ | *x*_2_ |
| A20 | 0.58 | -6.7 | 0.53 | -7.3 | 0.54 | -5.1 | 0.64 | -8.5 | 0.55 | -5.8 | 0.60 | -6.8 | 0.58 | -6.0 | 0.60 | -6.5 | 0.68 | -6.5 | 0.47 | -6.5 | 0.40 | -5.8 | 0.56 | -5.6 |
| ACE | 0.91 | -7.7 | 0.91 | -8.8 | 0.88 | -5.8 | 0.77 | -10.6 | 0.67 | -6.4 | 0.74 | -8.2 | 0.71 | -6.7 | 0.70 | -7.4 | 0.76 | -6.8 | 0.51 | -6.8 | 0.55 | -6.9 | 0.63 | -6.7 |
| AChE | 0.69 | -6.8 | 0.36 | -7.8 | 0.68 | -5.9 | 0.73 | -9.5 | 0.68 | -6.7 | 0.67 | -8.6 | 0.57 | -6.4 | 0.67 | -7.4 | 0.70 | -6.4 | 0.58 | -8.6 | 0.55 | -6.9 | 0.61 | -6.7 |
| ADAM10 | 0.43 | -6.2 | 0.42 | -6.2 | 0.45 | -4.6 | 0.50 | -7.1 | 0.37 | -4.9 | 0.51 | -6.1 | 0.45 | -5.7 | 0.49 | -6.9 | 0.47 | -6.2 | 0.43 | -6.3 | 0.39 | -5.7 | 0.45 | -6.5 |
| ADAM17 | 0.58 | -6.6 | 0.52 | -7.7 | 0.58 | -5.7 | 0.58 | -8.6 | 0.52 | -5.5 | 0.55 | -6.5 | 0.55 | -5.8 | 0.53 | -7.1 | 0.57 | -6.1 | 0.47 | -6.4 | 0.40 | -6.7 | 0.50 | -6.6 |
| APOE2 | 0.44 | -5.8 | 0.35 | -6.4 | 0.48 | -4.8 | 0.56 | -7.9 | 0.40 | -5.5 | 0.53 | -6.0 | 0.53 | -5.1 | 0.52 | -6.1 | 0.51 | -5.4 | 0.35 | -6.3 | 0.41 | -5.7 | 0.46 | -5.4 |
| APOE3 | 0.41 | -5.9 | 0.34 | -6.8 | 0.43 | -5.0 | 0.50 | -8.2 | 0.38 | -5.2 | 0.48 | -6.4 | 0.50 | -5.1 | 0.45 | -6.2 | 0.48 | -5.8 | 0.34 | -6.1 | 0.38 | -5.6 | 0.44 | -5.4 |
| APOE4 | 0.43 | -5.6 | 0.34 | -6.2 | 0.46 | -4.7 | 0.53 | -7.4 | 0.37 | -4.9 | 0.50 | -6.0 | 0.52 | -5.0 | 0.48 | -5.7 | 0.53 | -4.9 | 0.34 | -5.6 | 0.41 | -5.3 | 0.47 | -5.0 |
| BACE1 | 0.62 | -7.0 | 0.41 | -7.9 | 0.60 | -5.2 | 0.67 | -9.4 | 0.60 | -5.6 | 0.66 | -7.5 | 0.58 | -6.2 | 0.62 | -7.1 | 0.68 | -6.0 | 0.56 | -6.9 | 0.54 | -6.5 | 0.61 | -6.1 |
| BCHE | 0.63 | -7.5 | 0.43 | -8.4 | 0.62 | -5.3 | 0.65 | -10.4 | 0.65 | -6.3 | 0.66 | -8.3 | 0.52 | -6.4 | 0.61 | -7.6 | 0.67 | -6.9 | 0.56 | -7.3 | 0.51 | -6.4 | 0.62 | -6.8 |
| casp-6 | 0.56 | -6.6 | 0.51 | -7.9 | 0.50 | -5.5 | 0.65 | -9.2 | 0.39 | -6.2 | 0.62 | -7.7 | 0.57 | -6.2 | 0.58 | -6.7 | 0.59 | -6.4 | 0.43 | -7.1 | 0.44 | -6.5 | 0.52 | -6.5 |
| Cath- D | 0.44 | -7.5 | 0.34 | -7.7 | 0.44 | -5.3 | 0.62 | -9.4 | 0.43 | -6.0 | 0.54 | -7.3 | 0.53 | -6.5 | 0.55 | -7.2 | 0.58 | -6.2 | 0.35 | -6.0 | 0.40 | -7.0 | 0.49 | -6.0 |
| CDK5 | 0.54 | -7.1 | 0.41 | -7.5 | 0.53 | -5.5 | 0.62 | -8.1 | 0.55 | -5.6 | 0.64 | -8.2 | 0.54 | -5.9 | 0.61 | -7.2 | 0.63 | -5.4 | 0.47 | -6.2 | 0.49 | -6.6 | 0.53 | -6.5 |
| ChAT | 0.66 | -7.1 | 0.61 | -7.3 | 0.63 | -5.3 | 0.72 | -8.9 | 0.66 | -5.8 | 0.71 | -7.6 | 0.66 | -6.3 | 0.68 | -7.1 | 0.71 | -6.3 | 0.58 | -7.0 | 0.53 | -6.4 | 0.62 | -6.6 |
| CHRM2 | 0.63 | -7.7 | 0.48 | -8.0 | 0.59 | -5.9 | 0.75 | -9.2 | 0.60 | -6.5 | 0.88 | -8.3 | 0.88 | -6.2 | 0.64 | -8.1 | 0.70 | -6.4 | 0.52 | -7.9 | 0.50 | -6.8 | 0.57 | -6.7 |
| CHRM3 | 0.48 | -6.1 | 0.36 | -6.8 | 0.47 | -4.8 | 0.55 | -8.6 | 0.46 | -5.4 | 0.61 | -7.0 | 0.61 | -5.2 | 0.50 | -6.5 | 0.52 | -6.1 | 0.34 | -6.4 | 0.45 | -5.6 | 0.44 | -5.7 |
| CHRNA1 | 0.32 | -4.9 | 0.31 | -5.9 | 0.31 | -4.2 | 0.31 | -7.9 | 0.31 | -4.6 | 0.31 | -6.4 | 0.31 | -5.1 | 0.31 | -5.4 | 0.31 | -5.1 | 0.31 | -5.5 | 0.31 | -5.1 | 0.31 | -5.8 |
| CHRNA4 | 0.54 | -8.4 | 0.54 | -9.5 | 0.54 | -5.6 | 0.45 | -11.4 | 0.55 | -6.4 | 0.51 | -9.0 | 0.48 | -6.8 | 0.50 | -8.0 | 0.55 | -6.5 | 0.52 | -8.2 | 0.50 | -7.0 | 0.50 | -6.6 |
| CHRNA7 | 0.51 | -6.2 | 0.51 | -7.5 | 0.53 | -5.2 | 0.58 | -9.5 | 0.48 | -6.3 | 0.56 | -8.3 | 0.56 | -6.0 | 0.55 | -7.0 | 0.56 | -6.0 | 0.44 | -7.1 | 0.45 | -6.4 | 0.49 | -6.0 |
| CHRNA9 | 0.47 | -7.5 | 0.44 | -7.4 | 0.45 | -5.2 | 0.62 | -9.8 | 0.57 | -7.2 | 0.56 | -8.3 | 0.50 | -6.6 | 0.57 | -6.9 | 0.57 | -7.1 | 0.43 | -8.0 | 0.40 | -7.4 | 0.47 | -7.5 |
| CHRNB2 | 0.59 | -6.3 | 0.55 | -6.5 | 0.59 | -5.0 | 0.53 | -8.8 | 0.33 | -6.2 | 0.53 | -5.1 | 0.55 | -7.0 | 0.53 | -6.7 | 0.60 | -5.7 | 0.53 | -7.1 | 0.53 | -6.5 | 0.53 | -6.2 |
| CHRNB4 | 0.32 | -7.2 | 0.31 | -8.4 | 0.32 | -5.1 | 0.32 | -11.3 | 0.32 | -6.1 | 0.32 | -7.4 | 0.31 | -6.6 | 0.32 | -6.2 | 0.31 | -6.9 | 0.31 | -6.9 | 0.32 | -6.9 | 0.31 | -6.9 |
| CHRNE | 0.45 | -7.1 | 0.35 | -7.3 | 0.44 | -5 | 0.69 | -10.5 | 0.48 | -5.8 | 0.65 | -7.5 | 0.65 | -5.7 | 0.59 | -6.6 | 0.65 | -6.8 | 0.38 | -6.4 | 0.47 | -6.3 | 0.47 | -5.9 |
| COX | 0.68 | -8.4 | 0.65 | -9.3 | 0.66 | -6.1 | 0.77 | -10.0 | 0.66 | -7.1 | 0.72 | -9.1 | 0.74 | -7.3 | 0.63 | -7.9 | 0.79 | -6.8 | 0.55 | -7.4 | 0.55 | -6.7 | 0.69 | -7.0 |
| CYLD | 0.49 | -6.4 | 0.38 | -6.8 | 0.52 | -4.6 | 0.60 | -7.9 | 0.47 | -4.9 | 0.55 | -6.7 | 0.54 | -6.0 | 0.55 | -6.5 | 0.62 | -5.6 | 0.38 | -6.4 | 0.45 | -5.8 | 0.53 | -5.9 |
| CYP1A1 | 0.67 | -6.8 | 0.56 | -7.3 | 0.60 | -5.7 | 0.73 | -8.9 | 0.61 | -5.7 | 0.68 | -7.2 | 0.68 | -5.6 | 0.60 | -7.9 | 0.88 | -5.9 | 0.52 | -8.7 | 0.53 | -7.1 | 0.64 | -6.3 |
| DR6 | 0.41 | -5.5 | 0.37 | -6.5 | 0.41 | -4.4 | 0.43 | -7.1 | 0.42 | -4.7 | 0.45 | -5.3 | 0.42 | -4.5 | 0.45 | -5.5 | 0.44 | -4.7 | 0.36 | -5.2 | 0.39 | -5.1 | 0.44 | -5.0 |
| ECE | 0.62 | -7.9 | 0.61 | -8.8 | 0.62 | -6.0 | 0.68 | -10.0 | 0.55 | -6.2 | 0.66 | -8.4 | 0.64 | -6.8 | 0.62 | -7.4 | 0.69 | -6.9 | 0.50 | -7.1 | 0.50 | -6.7 | 0.56 | -7.4 |
| EphB2 | 0.46 | -6.3 | 0.41 | -7.0 | 0.47 | -4.8 | 0.62 | -8.4 | 0.44 | -5.3 | 0.57 | -6.2 | 0.52 | -5.4 | 0.52 | -6.3 | 0.58 | -6.0 | 0.41 | -6.4 | 0.43 | -5.9 | 0.45 | -5.5 |
| Factor XI | 0.51 | -7.2 | 0.44 | -7.9 | 0.47 | -5.2 | 0.60 | -8.8 | 0.38 | -5.5 | 0.54 | -7.4 | 0.51 | -6.4 | 0.53 | -6.6 | 0.59 | -6.4 | 0.35 | -6.7 | 0.46 | -6.0 | 0.86 | -6.5 |
| Fyn | 0.46 | -6.0 | 0.43 | -6.8 | 0.49 | -5.3 | 0.51 | -8.1 | 0.38 | -5.2 | 0.47 | -6.6 | 0.46 | -5.5 | 0.48 | -6.3 | 0.51 | -5.8 | 0.43 | -5.9 | 0.44 | -5.9 | 0.44 | -5.9 |
| GSK3β | 0.59 | -7.1 | 0.43 | -8.5 | 0.58 | -5.3 | 0.68 | -9.4 | 0.64 | -6.1 | 0.63 | -8.0 | 0.61 | -6.2 | 0.62 | -7.2 | 0.66 | -6.4 | 0.44 | -7.2 | 0.50 | -6.6 | 0.60 | -6.3 |
| IDE | 0.71 | -7.3 | 0.45 | -8.4 | 0.71 | -6.0 | 0.74 | -10.6 | 0.72 | -6.5 | 0.71 | -8.2 | 0.69 | -6.1 | 0.67 | -7.4 | 0.77 | -6.7 | 0.58 | -7.8 | 0.56 | -7.1 | 0.55 | -7.0 |
| IKK1,IKK2 | 0.31 | -5.0 | 0.31 | -6.2 | 0.33 | -4.5 | 0.33 | -7.1 | 0.31 | -4.8 | 0.33 | -6.3 | 0.31 | -5.5 | 0.33 | -5.1 | 0.31 | -5.3 | 0.31 | -5.8 | 0.32 | -4.9 | 0.32 | -4.8 |
| InSP3R | 0.53 | -6.3 | 0.43 | -7.1 | 0.49 | -5.1 | 0.62 | -8.0 | 0.50 | -5.4 | 0.58 | -6.8 | 0.51 | -5.8 | 0.60 | -6.3 | 0.61 | -5.5 | 0.41 | -6.5 | 0.42 | -5.9 | 0.53 | -5.8 |
| LilrB2 | 0.46 | -7.5 | 0.43 | -7.8 | 0.46 | -5.4 | 0.57 | -9.5 | 0.38 | -5.9 | 0.56 | -7.7 | 0.46 | -6.4 | 0.57 | -7.5 | 0.56 | -6.4 | 0.40 | -6.5 | 0.45 | -6.1 | 0.50 | -6.3 |
| LOX | 0.69 | -6.9 | 0.64 | -8.8 | 0.68 | -5.2 | 0.70 | -9.2 | 0.53 | -5.9 | 0.70 | -7.8 | 0.68 | -6.3 | 0.65 | -7.7 | 0.74 | -6.7 | 0.49 | -7.4 | 0.50 | -6.6 | 0.61 | -6.8 |
| MARK, | 0.56 | -7.0 | 0.54 | -8.1 | 0.60 | -5.5 | 0.68 | -9.8 | 0.56 | -6.0 | 0.66 | -7.3 | 0.57 | -6.3 | 0.68 | -6.6 | 0.67 | -6.5 | 0.35 | -6.7 | 0.48 | -6.3 | 0.59 | -6.4 |
| MM-2 | 0.63 | -7.5 | 0.47 | -8.8 | 0.61 | -5.9 | 0.66 | -10.4 | 0.53 | -6.1 | 0.70 | -7.5 | 0.66 | -6.6 | 0.70 | -7.5 | 0.73 | -6.9 | 0.53 | -7.9 | 0.54 | -6.7 | 0.58 | -7.1 |
| MM-9 | 0.50 | -7.4 | 0.40 | -8.3 | 0.52 | -5.7 | 0.61 | -10.2 | 0.50 | -6.4 | 0.58 | -8.0 | 0.55 | -6.5 | 0.58 | -7.2 | 0.61 | -6.7 | 0.43 | -7.7 | 0.50 | -7.4 | 0.52 | -7.2 |
| NEMO | 0.33 | -5.0 | 0.31 | -6.1 | 0.33 | -3.9 | 0.33 | -6.8 | 0.31 | -4.1 | 0.33 | -5.3 | 0.34 | -4.4 | 0.33 | -5.7 | 0.33 | -4.5 | 0.31 | -5.3 | 0.33 | -4.6 | 0.31 | -4.7 |
| NEP | 0.70 | -7.1 | 0.67 | -7.8 | 0.69 | -5.6 | 0.70 | -9.3 | 0.69 | -6.4 | 0.70 | -8.1 | 0.68 | -6.5 | 0.68 | -7.1 | 0.70 | -6.3 | 0.57 | -7.3 | 0.52 | -6.7 | 0.60 | -6.9 |
| NMDAR | 0.49 | -6.4 | 0.46 | -7.3 | 0.44 | -5.0 | 0.49 | -8.3 | 0.47 | -5.9 | 0.47 | -7.4 | 0.42 | -5.4 | 0.51 | -6.4 | 0.50 | -5.7 | 0.39 | -6.6 | 0.45 | -6.4 | 0.47 | -7.0 |
| PKC | 0.44 | -6.2 | 0.38 | -6.9 | 0.43 | -4.5 | 0.50 | -7.9 | 0.37 | -5.0 | 0.50 | -6.3 | 0.43 | -5.1 | 0.48 | -6.1 | 0.50 | -5.4 | 0.36 | -6.3 | 0.40 | -5.8 | 0.43 | -5.4 |
| PLA2 | 0.45 | -6.9 | 0.39 | -7.8 | 0.47 | -5.4 | 0.62 | -8.9 | 0.45 | -6.4 | 0.60 | -7.1 | 0.55 | -6.3 | 0.53 | -7.3 | 0.59 | -6.5 | 0.42 | -7.3 | 0.77 | -6.6 | 0.47 | -6.5 |
| PLC | 0.56 | -7.5 | 0.51 | -8.8 | 0.48 | -6.4 | 0.49 | -9.2 | 0.52 | -6.9 | 0.60 | -7.1 | 0.60 | -6.3 | 0.53 | -8.0 | 0.58 | -6.7 | 0.46 | -7.3 | 0.42 | -7.2 | 0.50 | -6.6 |
| PP2A | 0.54 | -7.3 | 0.42 | -8.0 | 0.60 | -5.5 | 0.63 | -9.3 | 0.57 | -6.4 | 0.58 | -6.9 | 0.63 | -6.2 | 0.58 | -7.2 | 0.65 | -6.7 | 0.41 | -7.2 | 0.47 | -6.5 | 0.53 | -6.7 |
| PrPC | 0.38 | -5.5 | 0.35 | -6.5 | 0.37 | -4.4 | 0.44 | -7.1 | 0.35 | -4.9 | 0.41 | -6.3 | 0.43 | -5.4 | 0.40 | -5.7 | 0.44 | -5.0 | 0.34 | -5.5 | 0.37 | -5.3 | 0.38 | -5.2 |
| PS1 | 0.39 | -6.0 | 0.36 | -6.8 | 0.39 | -4.5 | 0.49 | -7.6 | 0.38 | -5.0 | 0.57 | -5.8 | 0.50 | -5.3 | 0.43 | -5.7 | 0.48 | -5.6 | 0.34 | -6.6 | 0.40 | -6.8 | 0.38 | -5.3 |

The simulation of IC50 data

| **Figure A**. The picture of the inhibition rate for Bacitracin A inhibiting IDE | **Figure B**. The picture of the inhibition rate for 5-(N,N-Dimethyl) amiloride hydrochloride inhibiting IDE | **Figure C**. The picture of the inhibition rate for Amiloride HCl dehydrate inhibiting IDE |
| --- | --- | --- |
| **Figure D**. The picture of the inhibition rate for DL-Thiophan inhibiting NEP | **Figure E**. The picture of the inhibition rate for 5-(N,N-Dimethyl) amiloride hydrochloride inhibiting NEP | **Figure F**. The picture of the inhibition rate for Amiloride HCl dehydrate inhibiting NEP |

**Supplemental References**

1. Haass C, Selkoe, DJ. Cellular processing of beta-amyloid precursor protein and the genesis of amyloid beta-peptide. Cell 1993; 75 (4): 1039-1042.
2. Ulrike CM, Hui Z. Physiological functions of APP family proteins. Cold Spring Harb Perspect Med. 2012 Feb; 2(2): a006288
3. [Lazarov O](http://www.ncbi.nlm.nih.gov/pubmed/?term=Lazarov O%5BAuthor%5D&cauthor=true&cauthor_uid=22675290), [Demars MP](http://www.ncbi.nlm.nih.gov/pubmed/?term=Demars MP%5BAuthor%5D&cauthor=true&cauthor_uid=22675290). All in the family: how the APPs regulate neurogenesis. Front Neurosci. 2012 Jun 4; 6:81. doi: 10.3389/fnins.2012.00081. eCollection 2012.
4. Reinhard C, Hébert SS, De Strooper B.The amyloid-beta precursor protein: integrating structure with biological function. EMBO J. 2005 Dec 7; 24(23):3996-4006.
5. Iwata N, Tsubuki S, Takaki Y, [Shirotani K](http://www.ncbi.nlm.nih.gov/pubmed?term=Shirotani K%5BAuthor%5D&cauthor=true&cauthor_uid=11375493), [Lu B](http://www.ncbi.nlm.nih.gov/pubmed?term=Lu B%5BAuthor%5D&cauthor=true&cauthor_uid=11375493), [Gerard NP](http://www.ncbi.nlm.nih.gov/pubmed/?term=Gerard NP%5BAuthor%5D&cauthor=true&cauthor_uid=11375493), et al. Metabolic regulation of brain Abeta by neprilysin. Science. 200; 1292: 1550–1552.
6. Eckman EA, Reed DK, Eckman CB. Degradation of the Alzheimer’s amyloid beta peptide by endothelin-convertingenzyme. The Journal of Biological Chemistry. 2001; 276: 24540–24548.
7. Qiu WQ, Walsh DM, Ye Z, [Vekrellis K](http://www.ncbi.nlm.nih.gov/pubmed?term=Vekrellis K%5BAuthor%5D&cauthor=true&cauthor_uid=9830016), [Zhang J](http://www.ncbi.nlm.nih.gov/pubmed?term=Zhang J%5BAuthor%5D&cauthor=true&cauthor_uid=9830016), [Podlisny MB](http://www.ncbi.nlm.nih.gov/pubmed/?term=Podlisny MB%5BAuthor%5D&cauthor=true&cauthor_uid=9830016), et al. Insulin-degrading enzyme regulates extracellular levels of amyloid beta-protein by degradation. The Journal of Biological Chemistry. 1998; 273: 32730–32738.
8. Hu J, Igarashi A, Kamata M, Nakagawa H. Angiotensin-converting enzyme degrades Alzheimer amyloid beta-peptide (A beta); retards A beta aggregation, deposition, ﬁbrilformation; and inhibits cytotoxicity. The Journal of Biological Chemistry. 2001; 276: 47863–47868.
9. McDermott JR, Gibson AM. Degradation of Alzheimer’s β-amyloid protein by human cathepsin D. Neuroreport. 1996; 7: 2163-2166.
10. Yamada T, Miyazaki K, Koshikawa N, Takahashi M, Akatsu H, [Yamamoto T](http://www.ncbi.nlm.nih.gov/pubmed/?term=Yamamoto T%5BAuthor%5D&cauthor=true&cauthor_uid=7538720). Selective localization of gelatinase A, an enzyme degrading beta-amyloid protein, in white matter microglia and in Schwann cells. Acta Neuropathologica (Berl). 1995; 89: 199–203.
11. Backstrom JR, Lim GP, Cullen MJ, Tokes ZA. Matrixmetalloproteinase-9 (MMP-9) is synthesized in neurons of the human hippocampus and is capable of degrading the amyloid-beta peptide (1-40). The Journal of Neuroscience. 1996; 16: 7910–7919.
12. Saporito-Irwin SM, Van Nostrand WE. Coagulation factor XIa cleaves the RHDS sequence and abolishes the cell adhesive properties of the amyloid beta-protein. The Journal of Biological Chemistry. 1995; 270: 26265–26269.
13. Nikolaev A, McLaughlin T, O’Leary DD, Tessier-Lavigne M. APP binds DR6 to trigger axon pruning and neuron death via distinct caspases. Nature. 2009; 457: 981–990.
14. Bertrand E, Brouillet E, Caille I, Bouillot C, Cole GM, Prochiantz A, et al. A short cytoplasmic domain of the amyloid precursor protein induces apoptosis in vitro and in vivo. Mol Cell Neurosci 2001; 18: 503–511.
15. Lu DC, Soriano S, Bredesen DE, Koo EH. Caspase cleavage of the amyloid precursor protein modulates amyloid b-protein toxicity. J Neurochem 2003; 87: 733–741.
16. Park SA, Shaked GM, Bredesen DE, Koo, EH. Mechanism of cytotoxicity mediated by the C31 fragment of theamyloid precursor protein. BiochemBiophys Res Commun 2009; 388: 450–455.
17. [Texidó L](http://www.ncbi.nlm.nih.gov/pubmed?term=Texidó L%5BAuthor%5D&cauthor=true&cauthor_uid=21349580), [Martín-Satué M](http://www.ncbi.nlm.nih.gov/pubmed?term=Martín-Satué M%5BAuthor%5D&cauthor=true&cauthor_uid=21349580), [Alberdi E](http://www.ncbi.nlm.nih.gov/pubmed?term=Alberdi E%5BAuthor%5D&cauthor=true&cauthor_uid=21349580), [Solsona C](http://www.ncbi.nlm.nih.gov/pubmed?term=Solsona C%5BAuthor%5D&cauthor=true&cauthor_uid=21349580), [Matute C](http://www.ncbi.nlm.nih.gov/pubmed?term=Matute C%5BAuthor%5D&cauthor=true&cauthor_uid=21349580). Amyloid β peptide oligomers directly activate NMDA receptors. [Cell Calcium.](http://www.ncbi.nlm.nih.gov/pubmed/21349580/#%23) 2011; 49(3): 184-90.
18. Alberdi E, Sanchez-Gomez MV, Cavaliere F, Perez-Samartin A, Zugaza JL,  [Trullas R](http://www.ncbi.nlm.nih.gov/pubmed/?term=Trullas R%5BAuthor%5D&cauthor=true&cauthor_uid=20061018), et al. Amyloid beta oligomers induce Ca2+ dysregulation and neuronal death through activation of ionotropic glutamate receptors. Cell Calcium. 2010; 47: 264–272.
19. De Felice FG, Velasco PT, Lambert MP, Viola KL, Fernandez SJ, [Klein WL](http://www.ncbi.nlm.nih.gov/pubmed/?term=Klein WL%5BAuthor%5D&cauthor=true&cauthor_uid=17308309). Abeta oligomers induce neuronal oxidative stress through an NMDA receptor-dependent mechanism that is blocked by the Alzheimer’s drug memantine. J Biol Chem. 2007; 282: 11590–11601.
20. Cissé M, Halabisky B, Harris J, Devidze N, Dubal DB,  [Sun B](http://www.ncbi.nlm.nih.gov/pubmed/?term=Sun B%5BAuthor%5D&cauthor=true&cauthor_uid=21113149), et al. Reversing EphB2 depletion rescues cognitive functions in Alzheimer model. Nature. 2011; 469: 47–52.
21. [Kim T](http://www.ncbi.nlm.nih.gov/pubmed?term=Kim T%5BAuthor%5D&cauthor=true&cauthor_uid=24052308), [Vidal GS](http://www.ncbi.nlm.nih.gov/pubmed?term=Vidal GS%5BAuthor%5D&cauthor=true&cauthor_uid=24052308), [Djurisic M](http://www.ncbi.nlm.nih.gov/pubmed?term=Djurisic M%5BAuthor%5D&cauthor=true&cauthor_uid=24052308), [William CM](http://www.ncbi.nlm.nih.gov/pubmed?term=William CM%5BAuthor%5D&cauthor=true&cauthor_uid=24052308), [Birnbaum ME](http://www.ncbi.nlm.nih.gov/pubmed?term=Birnbaum ME%5BAuthor%5D&cauthor=true&cauthor_uid=24052308), [Garcia KC](http://www.ncbi.nlm.nih.gov/pubmed/?term=Garcia KC%5BAuthor%5D&cauthor=true&cauthor_uid=24052308), et al. Human LilrB2 is a β-amyloid receptor and its murine homolog PirB regulates synaptic plasticity in an Alzheimer's model. [Science.](http://www.ncbi.nlm.nih.gov/pubmed/24052308) 2013 Sep 20; 341(6152): 1399-404.
22. Laurén J, Gimbel DA, Nygaard HB, Gilbert JW, Strittmatter SM. Cellular prion protein mediates impairment of synaptic plasticity by amyloid-beta oligomers. Nature. 2009; 457: 1128– 1132.
23. [Um JW](http://www.ncbi.nlm.nih.gov/pubmed?term=Um JW%5BAuthor%5D&cauthor=true&cauthor_uid=22820466), [Nygaard HB](http://www.ncbi.nlm.nih.gov/pubmed?term=Nygaard HB%5BAuthor%5D&cauthor=true&cauthor_uid=22820466), [Heiss JK](http://www.ncbi.nlm.nih.gov/pubmed?term=Heiss JK%5BAuthor%5D&cauthor=true&cauthor_uid=22820466), [Kostylev MA](http://www.ncbi.nlm.nih.gov/pubmed?term=Kostylev MA%5BAuthor%5D&cauthor=true&cauthor_uid=22820466), [Stagi M](http://www.ncbi.nlm.nih.gov/pubmed?term=Stagi M%5BAuthor%5D&cauthor=true&cauthor_uid=22820466), [Vortmeyer A](http://www.ncbi.nlm.nih.gov/pubmed/?term=Vortmeyer A%5BAuthor%5D&cauthor=true&cauthor_uid=22820466), et al. Alzheimer amyloid-β oligomer bound to postsynaptic prion protein activates Fyn to impair neurons. [Nat Neurosci.](http://www.ncbi.nlm.nih.gov/pubmed/?term=Um+JW%2C+et+al.+Nat.+Neurosci.+2012%3B+15%3A1227–1235) 2012 Sep; 15(9): 1227-35.
24. Bu G. Apolipoprotein E and its receptors in Alzheimer’s disease: pathways, pathogenesis and therapy. Nat. Rev. Neurosci. 2009; 10: 333–344.
25. Kim J,  [Castellano JM](http://www.ncbi.nlm.nih.gov/pubmed/?term=Castellano JM%5BAuthor%5D&cauthor=true&cauthor_uid=20005821), [Jiang H](http://www.ncbi.nlm.nih.gov/pubmed/?term=Jiang H%5BAuthor%5D&cauthor=true&cauthor_uid=20005821), [Basak JM](http://www.ncbi.nlm.nih.gov/pubmed/?term=Basak JM%5BAuthor%5D&cauthor=true&cauthor_uid=20005821), [Parsadanian M](http://www.ncbi.nlm.nih.gov/pubmed/?term=Parsadanian M%5BAuthor%5D&cauthor=true&cauthor_uid=20005821),  [Pham V](http://www.ncbi.nlm.nih.gov/pubmed/?term=Pham V%5BAuthor%5D&cauthor=true&cauthor_uid=20005821), [Mason SM](http://www.ncbi.nlm.nih.gov/pubmed/?term=Mason SM%5BAuthor%5D&cauthor=true&cauthor_uid=20005821), et al. Overexpression of low-density lipoprotein receptor in the brain markedly inhibits amyloid deposition and increases extracellular Aβ clearance. Neuron. 2009; 64: 632–644.
26. Xia M, Cheng X, Yi R, Gao D, Xiong J.The Binding Receptors of Aβ: an Alternative Therapeutic Target for Alzheimer's Disease. Mol Neurobiol. 2014 Dec 4.doi: 10.1007/s12035-014-8994-0.
27. Chen CH, Zhou W, Liu S, Deng Y, Cai F, Tone M, et al. Increased NF-κB signalling up-regulates BACE1 expression and its therapeutic potential in Alzheimer's disease. Int J Neuropsychopharmacol. 2012 Feb; 15(1): 77-90.
28. Perkins ND. Integrating cell-signalling pathways with NF-κB and IKK function.Nat. Rev. Mol. Cell Biol. 2007 January; 8 (1): 49–62
29. [Chami L](http://www.ncbi.nlm.nih.gov/pubmed/?term=Chami L%5BAuthor%5D&cauthor=true&cauthor_uid=22654105), [Buggia-Prévot V](http://www.ncbi.nlm.nih.gov/pubmed/?term=Buggia-Prévot V%5BAuthor%5D&cauthor=true&cauthor_uid=22654105), [Duplan E](http://www.ncbi.nlm.nih.gov/pubmed/?term=Duplan E%5BAuthor%5D&cauthor=true&cauthor_uid=22654105), [Delprete D](http://www.ncbi.nlm.nih.gov/pubmed/?term=Delprete D%5BAuthor%5D&cauthor=true&cauthor_uid=22654105), [Chami M](http://www.ncbi.nlm.nih.gov/pubmed/?term=Chami M%5BAuthor%5D&cauthor=true&cauthor_uid=22654105), [Peyron JF](http://www.ncbi.nlm.nih.gov/pubmed/?term=Peyron JF%5BAuthor%5D&cauthor=true&cauthor_uid=22654105),  [et](http://www.ncbi.nlm.nih.gov/pubmed/?term=Checler F%5BAuthor%5D&cauthor=true&cauthor_uid=22654105) al. Nuclear Factor-κB Regulates βAPP and Aβ and γ-Secretases Differently at Physiological and Supraphysiological Aβ Concentrations. [J Biol Chem.](http://www.ncbi.nlm.nih.gov/pubmed/?term=Nuclear+Factor-κB+Regulates+βAPP+and+Aβ+and+γ-Secretases+Differently+at+Physiological+and+Supraphysiological+Aβ+Concentrations.) 2012 Jul 13; 287(29): 24573-84.
30. Barger S, Horster D, Furukawa K, Goodman Y, Krieglstein J, Mattson M. Tumor necrosis factors α and β protect neurons against amyloid β -peptide toxicity: evidence for involvement of a κB-binding factor and attenuation of peroxide and Ca2+ accumula-tion. Proc. Natl. Acad. Sci. USA. 1995; 92: 9328–9332
31. Combs CK, Karlo JC, Kao SC, Landreth GE. Beta-Amyloid stimulation of microglia and monocytes results in TNF alpha-dependent expression of inducible nitric oxide synthase and neuronal apoptosis. J Neurosci. 2001; 21: 1179-88.
32. Shembade N, Ma A, Harhaj EW. Inhibition of NF-kB signaling by A20 through disruption of ubiquitin enzyme complexes. Science. 2010 Feb 26; 327(5969):1135-9.
33. Takami Y, Nakagami H, Morishita R, Katsuya T, Cui TX, [Ichikawa T](http://www.ncbi.nlm.nih.gov/pubmed/?term=Ichikawa T%5BAuthor%5D&cauthor=true&cauthor_uid=17690318), et al. Ubiquitin carboxyl-terminal hydrolase L1, a novel deubiquitinating enzyme in the vasculature, attenuates NF-kappaB activation. Arterioscler Thromb Vasc Biol. 2007 Oct; 27(10): 2184-90.
34. Trompouki E, Hatzivassiliou E, Tsichritzis T, Farmer H, Ashworth A, Mosialos G. CYLD is a deubiquitinating enzyme that negatively regulates NF-kB activation by TNFR family members. Nature. 2003 Aug 14; 424(6950): 793-6.
35. Chen J, Zhou Y, Mueller-Steiner S, Chen LF, Kwon H, [Yi S](http://www.ncbi.nlm.nih.gov/pubmed/?term=Yi S%5BAuthor%5D&cauthor=true&cauthor_uid=16183991), et al. SIRT1 protects against microglia-dependent amyloid-beta toxicity through inhibiting NF-kappaB signaling. J Biol Chem. 2005 Dec 2; 280(48): 40364-74
36. Tolias KF, Cantley LC. Pathways for phosphoinositide synthesis. ChemPhys Lipids. 1999; 98: 69-77.
37. Hayley M, Perspicace S, Schulthess T, Seelig J. Calcium enhances the proteolytic activity of BACE1: An in vitro biophysical and biochemical characterization of the BACE1–calcium interaction. Biochimica et Biophysica Acta. 2009; 1788(9): 1933-1938
38. Alberts B, Lewis J, Raff M, Roberts K, Walter P. Molecular biology of the cell (4th ed.). New York: Garland Science; 2002.
39. Hung AY, Haass C, Nitsc RM, Qiu WQ, Citron M,  [Wurtman RJ](http://www.ncbi.nlm.nih.gov/pubmed/?term=Wurtman RJ%5BAuthor%5D&cauthor=true&cauthor_uid=8226807), [Growdon JH](http://www.ncbi.nlm.nih.gov/pubmed/?term=Growdon JH%5BAuthor%5D&cauthor=true&cauthor_uid=8226807), [Selkoe DJ](http://www.ncbi.nlm.nih.gov/pubmed/?term=Selkoe DJ%5BAuthor%5D&cauthor=true&cauthor_uid=8226807). Activation of protein kinase C inhibits cellular production of the amyloid beta-protein. J. Biol. Chem.1993; 268: 22959–22962.
40. Lin LL, Lin AY, Knopf JL. Cytosolic phospholipase A2 is coupled to hormonally regulated release of arachidonic acid, Proc Natl Acad Sci U S A. 1992; 89(13): 6147-6151.
41. Nishiyama M, Okamoto H, Watanabe T, Hori T, Hada T, [Ueda N](http://www.ncbi.nlm.nih.gov/pubmed/?term=Ueda N%5BAuthor%5D&cauthor=true&cauthor_uid=1548473), et al. Localization of arachidonate 12-lipoxygenase in canine brain tissues, J. Neurochem. 1992; 58(4): 1395-1400.
42. Roman RJ. P-450 metabolites of arachidonic acid in the control of cardiovascular function. Physiol Rev. 2002 Jan; 82(1): 131-85.
43. Amtul Z, Uhrig M, Wang L, Rozmahel RF, Beyreuther K. Detrimental effects of arachidonic acid and its metabolites in cellular and mouse models of Alzheimer's disease: Structural insight. Neurobiology of Aging. 2012; 33 (4): 831.e21–31
44. Camandola S, Leonarduzzi G, Musso T, Varesio L, [Carini R](http://www.ncbi.nlm.nih.gov/pubmed/?term=Carini R%5BAuthor%5D&cauthor=true&cauthor_uid=8954951), [Scavazza A](http://www.ncbi.nlm.nih.gov/pubmed/?term=Scavazza A%5BAuthor%5D&cauthor=true&cauthor_uid=8954951), et al. NF-kB is activated by arachidonic acid but not by eicosapentaenoic acid. Biochem Biophys Res Commun. 1996 Dec 13; 229(2): 643-7.
45. Namba Y, Tomonaga M, Kawasaki H, Otomo E, Ikeda K. Apolipoprotein E immunoreactivity in cerebral amyloid deposits and neurofibrillary tangles in Alzheimer’s disease and kuru plaque amyloid in Creutzfeldt–Jakob disease. Brain Res. 1991; 541: 163–166.
46. Corder EH, Saunders AM, Strittmatter WJ, Schmechel DE, Gaskell PC,  [Small GW](http://www.ncbi.nlm.nih.gov/pubmed/?term=Small GW%5BAuthor%5D&cauthor=true&cauthor_uid=8346443), et al. Gene dose of apolipoprotein E type 4 allele and the risk of Alzheimer’s disease in late onset families. Science. 1993; 261: 921–923.
47. [Kim J](http://www.ncbi.nlm.nih.gov/pubmed/?term=Kim J%5BAuthor%5D&cauthor=true&cauthor_uid=22159114), [Jiang H](http://www.ncbi.nlm.nih.gov/pubmed/?term=Jiang H%5BAuthor%5D&cauthor=true&cauthor_uid=22159114), [Park S](http://www.ncbi.nlm.nih.gov/pubmed/?term=Park S%5BAuthor%5D&cauthor=true&cauthor_uid=22159114), [Eltorai AE](http://www.ncbi.nlm.nih.gov/pubmed/?term=Eltorai AE%5BAuthor%5D&cauthor=true&cauthor_uid=22159114), [Stewart FR](http://www.ncbi.nlm.nih.gov/pubmed/?term=Stewart FR%5BAuthor%5D&cauthor=true&cauthor_uid=22159114), [Yoon H](http://www.ncbi.nlm.nih.gov/pubmed/?term=Yoon H%5BAuthor%5D&cauthor=true&cauthor_uid=22159114), et al. Haploinsufficiency of human APOE reduces amyloid deposition in a mouse model of amyloid-β amyloidosis. J. Neurosci. 2011; 31: 18007–18012.
48. Bien-Ly N, Gillespie AK, Walker D, Yoon SY, Huang Y. Reducing human apolipoprotein E levels attenuates age-dependent Aβ accumulation in mutant human amyloid precursor protein transgenic mice. J. Neurosci. 2012; 32: 4803–4811.
49. LaDu MJ, Falduto MT, Manelli AM, Reardon CA, Getz GS, Frail DE. Isoform-specific binding of apolipoprotein E to beta-amyloid. J Biol Chem. 1994; 269: 23403–23406.
50. Yang DS, Smith JD, Zhou Z, Gandy SE, Martins RN. Characterization of the binding of amyloid-beta peptide to cell culture-derived native apolipoprotein E2, E3, and E4 isoforms and to isoforms from human plasma. J Neurochem. 1997; 68: 721- 725.
51. Kanekiyo T, Zhang J, Liu Q, Liu CC, Zhang L, Bu G. Heparansulphate proteoglycan and the low-density lipoprotein receptor-related protein 1 constitute major pathways for neuronal amyloid-β uptake. J. Neurosci. 2011; 31: 1644–1651.
52. Zerbinatti CV, [Wozniak DF](http://www.ncbi.nlm.nih.gov/pubmed/?term=Wozniak DF%5BAuthor%5D&cauthor=true&cauthor_uid=14732699), [Cirrito J](http://www.ncbi.nlm.nih.gov/pubmed/?term=Cirrito J%5BAuthor%5D&cauthor=true&cauthor_uid=14732699), [Cam JA](http://www.ncbi.nlm.nih.gov/pubmed/?term=Cam JA%5BAuthor%5D&cauthor=true&cauthor_uid=14732699), [Osaka H](http://www.ncbi.nlm.nih.gov/pubmed/?term=Osaka H%5BAuthor%5D&cauthor=true&cauthor_uid=14732699), [Bales KR](http://www.ncbi.nlm.nih.gov/pubmed/?term=Bales KR%5BAuthor%5D&cauthor=true&cauthor_uid=14732699), et al. Increased soluble amyloid-β peptide and memory deficits in amyloid model mice overexpressing the low-density lipoprotein receptor-related protein. Proc. Natl Acad. Sci. USA 2004; 101: 1075–1080.
53. Nukina N, Ihara Y. One of the antigenic determinants of paired helical filaments is related to tau protein. J Biochem. 1986; 991: 1541–1544.
54. Kondo J, Honda T, Mori H, [Hamada Y](http://www.ncbi.nlm.nih.gov/pubmed/?term=Hamada Y%5BAuthor%5D&cauthor=true&cauthor_uid=2483105), [Miura R](http://www.ncbi.nlm.nih.gov/pubmed/?term=Miura R%5BAuthor%5D&cauthor=true&cauthor_uid=2483105), [Ogawara M](http://www.ncbi.nlm.nih.gov/pubmed/?term=Ogawara M%5BAuthor%5D&cauthor=true&cauthor_uid=2483105), et al. The carboxyl third of tau is tightly bound to paired helical filaments. Neuron.  1988 Nov; 1(9): 827–834.
55. Baum L, Hansen L, Masliah E, Saitoh T. Glycogen synthase kinase 3 alteration in Alzheimer disease is related to neuro?brillary tangle formation. Mol. Chem. Neuropathol. 1996; 29: 253–261.
56. Drewes G, Ebneth A, Preuss U, Mandelkow EM, Mandelkow E. MARK, a novel family of protein kinases that phosphorylate microtubule-associated proteins and trigger microtubule disruption. Cell. 1997; 89: 297–308.
57. Augustinack JC, Schneider A, Mandelkow EM, Hyman BT. Specific tau phosphorylation sites correlate with severity ofneuronal cytopathology in Alzheimer’s disease. ActaNeuropathol. (Berl.). 2002; 103: 26–35.
58. Li G, Yin H, Kuret J. Casein Kinase 1 Delta Phosphorylates Tau and Disrupts Its Binding to Microtubules. J Biol Chem. 2004; Apr 16;279(16):15938-45
59. [Liu F](http://www.ncbi.nlm.nih.gov/pubmed/?term=Liu F%5BAuthor%5D&cauthor=true&cauthor_uid=16262633), [Grundke-Iqbal I](http://www.ncbi.nlm.nih.gov/pubmed/?term=Grundke-Iqbal I%5BAuthor%5D&cauthor=true&cauthor_uid=16262633), [Iqbal K](http://www.ncbi.nlm.nih.gov/pubmed/?term=Iqbal K%5BAuthor%5D&cauthor=true&cauthor_uid=16262633),  [Gong CX](http://www.ncbi.nlm.nih.gov/pubmed/?term=Gong CX%5BAuthor%5D&cauthor=true&cauthor_uid=16262633). Contributions of protein phosphatases PP1, PP2A, PP2B and PP5 to the regulation of tau phosphorylation. European Journal of Neuroscience. 2005; 22: 1942–1950.
60. Woolf NJ. The critical role of cholinergic basal forebrain neurons in morphological change and memory encoding: a hypothesis. Neurobiol. Learn. Mem. 1996; 66: 258–266.
61. Augustinsson KB, Nachmansohn D. Distinction between acetylcholinesterase and other choline estersplitting enzymes. Science. 1949; 110: 98–99.
62. Li B,  [Stribley JA](http://www.ncbi.nlm.nih.gov/pubmed/?term=Stribley JA%5BAuthor%5D&cauthor=true&cauthor_uid=10936216), [Ticu A](http://www.ncbi.nlm.nih.gov/pubmed/?term=Ticu A%5BAuthor%5D&cauthor=true&cauthor_uid=10936216), [Xie W](http://www.ncbi.nlm.nih.gov/pubmed/?term=Xie W%5BAuthor%5D&cauthor=true&cauthor_uid=10936216), [Schopfer LM](http://www.ncbi.nlm.nih.gov/pubmed/?term=Schopfer LM%5BAuthor%5D&cauthor=true&cauthor_uid=10936216), [Hammond P](http://www.ncbi.nlm.nih.gov/pubmed/?term=Hammond P%5BAuthor%5D&cauthor=true&cauthor_uid=10936216), et al. Abundant tissue butyrylcholinesterase and its possible function in the acetylcholinesterase knockout mouse. J. Neurochem. 2000; 75: 1320–1331.
63. Takeshi K, Shun S. Alzheimer's disease and acetylcholine receptors. Acta Neurobiol Exp. 2004; 64: 99-105
64. [Qin K](http://www.ncbi.nlm.nih.gov/pubmed/?term=Qin K%5BAuthor%5D&cauthor=true&cauthor_uid=21873996), [Dong C](http://www.ncbi.nlm.nih.gov/pubmed/?term=Dong C%5BAuthor%5D&cauthor=true&cauthor_uid=21873996), [Wu G](http://www.ncbi.nlm.nih.gov/pubmed/?term=Wu G%5BAuthor%5D&cauthor=true&cauthor_uid=21873996),  [Lambert NA](http://www.ncbi.nlm.nih.gov/pubmed/?term=Lambert NA%5BAuthor%5D&cauthor=true&cauthor_uid=21873996). Inactive-state preassembly of Gq-coupled receptors and Gq heterotrimers. Nature Chemical Biology. 2011; 7 (11): 740–747.
65. Burford NT, Nahorski SR. Muscarinic m1 receptor-stimulated adenylate cyclase activity in Chinese hamster ovary cells is mediated by Gs alpha and is not a consequence of phosphoinositidase C activation. Biochem. J. 1996; 315 (Pt 3): 883–8.
66. Buxbaum JD, [Gandy SE](http://www.ncbi.nlm.nih.gov/pubmed/?term=Gandy SE%5BAuthor%5D&cauthor=true&cauthor_uid=2116015), [Cicchetti P](http://www.ncbi.nlm.nih.gov/pubmed/?term=Cicchetti P%5BAuthor%5D&cauthor=true&cauthor_uid=2116015), [Ehrlich ME](http://www.ncbi.nlm.nih.gov/pubmed/?term=Ehrlich ME%5BAuthor%5D&cauthor=true&cauthor_uid=2116015), [Czernik AJ](http://www.ncbi.nlm.nih.gov/pubmed/?term=Czernik AJ%5BAuthor%5D&cauthor=true&cauthor_uid=2116015), [Fracasso RP](http://www.ncbi.nlm.nih.gov/pubmed/?term=Fracasso RP%5BAuthor%5D&cauthor=true&cauthor_uid=2116015), et al. Processing of Alzheimer beta/A4 amyloid precursor protein: modulation by agents that regulate protein phosphorylation. Proc. Natl Acad. Sci. USA 1990; 87: 6003–6006.
67. Thathiah A, De Strooper B. The role of G protein-coupled receptors in the pathology of Alzheimer's disease. Nat Rev Neurosci. 2011 Feb; 12(2): 73-87.
68. Nitsch RM, Slack BE, Wurtman RJ, Growdon JH. Release of Alzheimer amyloid precursor derivatives stimulated by activation of muscarinic acetylcholine receptors. Science. 1992; 258: 304–307.
69. Buxbaum JD, [Oishi M](http://www.ncbi.nlm.nih.gov/pubmed/?term=Oishi M%5BAuthor%5D&cauthor=true&cauthor_uid=1359534), [Chen HI](http://www.ncbi.nlm.nih.gov/pubmed/?term=Chen HI%5BAuthor%5D&cauthor=true&cauthor_uid=1359534), [Pinkas-Kramarski R](http://www.ncbi.nlm.nih.gov/pubmed/?term=Pinkas-Kramarski R%5BAuthor%5D&cauthor=true&cauthor_uid=1359534), [Jaffe EA](http://www.ncbi.nlm.nih.gov/pubmed/?term=Jaffe EA%5BAuthor%5D&cauthor=true&cauthor_uid=1359534), [Gandy SE](http://www.ncbi.nlm.nih.gov/pubmed/?term=Gandy SE%5BAuthor%5D&cauthor=true&cauthor_uid=1359534), et al. Cholinergic agonists and interleukin 1 regulate processing and secretion of the Alzheimer beta/A4 amyloid protein precursor. Proc. Natl Acad. Sci. USA 1992; 89: 10075–10078.
70. Thathiah A, De Strooper B. G protein-coupled receptors, cholinergic dysfunction, and Abeta toxicity in Alzheimer’s disease. Sci. Signal. 2009; 2: re8.
71. Sadot E, [Sadot E](http://www.ncbi.nlm.nih.gov/pubmed/?term=Sadot E%5BAuthor%5D&cauthor=true&cauthor_uid=8592166), [Gurwitz D](http://www.ncbi.nlm.nih.gov/pubmed/?term=Gurwitz D%5BAuthor%5D&cauthor=true&cauthor_uid=8592166), [Barg J](http://www.ncbi.nlm.nih.gov/pubmed/?term=Barg J%5BAuthor%5D&cauthor=true&cauthor_uid=8592166), [Behar L](http://www.ncbi.nlm.nih.gov/pubmed/?term=Behar L%5BAuthor%5D&cauthor=true&cauthor_uid=8592166), [Ginzburg I](http://www.ncbi.nlm.nih.gov/pubmed/?term=Ginzburg I%5BAuthor%5D&cauthor=true&cauthor_uid=8592166), et al. Activation of m1 muscarinic acetylcholine receptor regulates tau phosphorylation in transfected PC12 cells. J. Neurochem. 1996; 66: 877–880.
72. Forlenza OV,  [Spink JM](http://www.ncbi.nlm.nih.gov/pubmed/?term=Spink JM%5BAuthor%5D&cauthor=true&cauthor_uid=11129110), [Dayanandan R](http://www.ncbi.nlm.nih.gov/pubmed/?term=Dayanandan R%5BAuthor%5D&cauthor=true&cauthor_uid=11129110), [Anderton BH](http://www.ncbi.nlm.nih.gov/pubmed/?term=Anderton BH%5BAuthor%5D&cauthor=true&cauthor_uid=11129110), [Olesen OF](http://www.ncbi.nlm.nih.gov/pubmed/?term=Olesen OF%5BAuthor%5D&cauthor=true&cauthor_uid=11129110),  [Lovestone S](http://www.ncbi.nlm.nih.gov/pubmed/?term=Lovestone S%5BAuthor%5D&cauthor=true&cauthor_uid=11129110). Muscarinic agonists reduce tau phosphorylation in non-neuronal cells via GSK-3beta inhibition and in neurons [J]. J Neural lyansm. 2000; 107(10): 1201-1212
73. Gray R, Rajan AS, Radcliffe KA, Yakehiro M, Dani JA. Hippocampal synaptic transmission enhanced by low concentrations of nicotine. Nature. 1996; 383(6602): 713–6.
74. Ji D, Lape R, Dani JA. Timing and location of nicotinic activity enhances or depresses hippocampal synaptic plasticity. Neuron. 2001; 31(1): 131–41.
75. Wilson AL, Langley LK, Monley J, Bauer T, Rottunda S, McFalls E, et al. Nicotine patches in Alzheimer’s disease: pilot study on learning, memory, and safety. Pharmacol Biochem Behav. 1995; 51(2–3): 509–14.
76. Lopez-Arrieta JM, Rodriguez JL, Sanz F. Efficacy and safety of nicotine on Alzheimer’s disease patients. Cochrane Database Syst Rev. 2001; 2: CD001749.
77. Li Y, Papke RL, Martin EJ, He YJ, Millard WJ, Meyer EM. Characterization of the neuroprotective and toxic effects of alpha7 nicotinic receptor activation in PC12 cells. Brain Res. 1999; 816: 225–230.
78. Wang HY, Lee DH, D’Andrea MR, Peterson PA, Shank RP, Reitz AB. Beta-amyloid (1-42) binds to alpha7 nicotinic acetylcholine receptor with high affinity. Implications for Alzheimer’s disease pathology. J Biol Chem. 2000; 275(8): 5626–32.
79. Wang HY, Lee DH, Davis CB, Shank RP. Amyloid peptide abeta (1-42) binds selectively and with picomolar affinity to alpha7 nicotinic acetylcholine receptors. J Neurochem. 2000; 75(3): 1155–61.
80. Wang HY, [Li W](http://www.ncbi.nlm.nih.gov/pubmed/?term=Li W%5BAuthor%5D&cauthor=true&cauthor_uid=12801934), [Benedetti NJ](http://www.ncbi.nlm.nih.gov/pubmed/?term=Benedetti NJ%5BAuthor%5D&cauthor=true&cauthor_uid=12801934), [Lee, DH](http://www.ncbi.nlm.nih.gov/pubmed/?term=Lee DH%5BAuthor%5D&cauthor=true&cauthor_uid=12801934). Alpha7 nicotinic acetylcholine receptors mediate beta -amyloid peptide-induced Tau protein phosphorylation. J. B iol Chem. 2003; 278 (34): 31547–31553
81. Dziewczapolski G, Glogowski CM, Masliah E, Heinemann SF. Deletion of the alpha 7 nicotinic acetylcholine receptor gene improves cognitive deficits and synaptic pathology in a mouse model of Alzheimer’s disease. J Neurosci. 2009; 29: 8805–15.
82. Yoshikawa H, Kurokawa M, Ozaki N, [Nara K](http://www.ncbi.nlm.nih.gov/pubmed/?term=Nara K%5BAuthor%5D&cauthor=true&cauthor_uid=16968406), [Atou K](http://www.ncbi.nlm.nih.gov/pubmed/?term=Atou K%5BAuthor%5D&cauthor=true&cauthor_uid=16968406), [Takada E](http://www.ncbi.nlm.nih.gov/pubmed/?term=Takada E%5BAuthor%5D&cauthor=true&cauthor_uid=16968406), et al. Nicotine inhibits the production of proinflammatory mediators in human monocytes by suppression of I-KappaB phosphorylation and nuclear factor- KappaB transcriptional activity through nicotinic acetylcholine receptor alpha7. Clinical and Experimental Immunology. 2006; 146(1): 116-123.
83. [Götz J](http://www.ncbi.nlm.nih.gov/pubmed/?term=Götz J%5BAuthor%5D&cauthor=true&cauthor_uid=11520988), [Chen F](http://www.ncbi.nlm.nih.gov/pubmed/?term=Chen F%5BAuthor%5D&cauthor=true&cauthor_uid=11520988), [van Dorpe J](http://www.ncbi.nlm.nih.gov/pubmed/?term=van Dorpe J%5BAuthor%5D&cauthor=true&cauthor_uid=11520988),  [Nitsch RM](http://www.ncbi.nlm.nih.gov/pubmed/?term=Nitsch RM%5BAuthor%5D&cauthor=true&cauthor_uid=11520988). Formation of neurofibrillary tangles in P301L tau transgenic mice induced by Aβ 42 fibrils. Science. 2001; 293(5534): 1491–1495.
84. Busciglio J, Lorenzo A，Yeh J, [Yankner B A](http://www.ncbi.nlm.nih.gov/pubmed/?term=Yankner BA%5BAuthor%5D&cauthor=true&cauthor_uid=7718249). Beta-amyloid fibrils induce tau phosphorylation and loss of microtubule binding. Neuron. 1995; 14: 879-888．
85. Zempel H, Thies E, Mandelkow E, Mandelkow EM. Aβ oligomers cause localized Ca2+ elevation, missorting of endogenous Tau into dendrites, Tau phosphorylation, and destruction of microtubules and spines. [J Neurosci.](http://www.ncbi.nlm.nih.gov/pubmed/?term=Aβ+oligomers+cause+localized+Ca2%2B+elevation%2C+missorting+of+endogenous+Tau+into+dendrites%2C+Tau+phosphorylation%2C+and+destruction+of+microtubules+and+spines.) 2010 Sep 8; 30(36): 11938-50.
86. Roberson ED，Scearce-Levie K，Palop JJ，[Yan F](http://www.ncbi.nlm.nih.gov/pubmed/?term=Yan F%5BAuthor%5D&cauthor=true&cauthor_uid=17478722), [Cheng IH](http://www.ncbi.nlm.nih.gov/pubmed/?term=Cheng IH%5BAuthor%5D&cauthor=true&cauthor_uid=17478722), [Wu T](http://www.ncbi.nlm.nih.gov/pubmed/?term=Wu T%5BAuthor%5D&cauthor=true&cauthor_uid=17478722), et al. Reducing endogenous tau ameliorates amyloid beta-induced deficits in an Alzheimer’s disease mouse model. Science. 2007; 316: 750-754．
87. Lttner LM, Ke YD, Delerue F, [Bi M](http://www.ncbi.nlm.nih.gov/pubmed/?term=Bi M%5BAuthor%5D&cauthor=true&cauthor_uid=20655099), [Gladbach A](http://www.ncbi.nlm.nih.gov/pubmed/?term=Gladbach A%5BAuthor%5D&cauthor=true&cauthor_uid=20655099), [van Eersel J](http://www.ncbi.nlm.nih.gov/pubmed/?term=van Eersel J%5BAuthor%5D&cauthor=true&cauthor_uid=20655099), et al. Dendritic function of tau mediates amyloid-beta toxicity in Alzheimer’s disease mouse models. Cell. 2010; 142: 387-397.
